# Exceptionally fast radiative decay of a dinuclear platinum complex through thermally activated delayed fluorescence†

**DOI:** 10.1039/d1sc00160d

**Published:** 2021-03-22

**Authors:** Piotr Pander, Ruth Daniels, Andrey V. Zaytsev, Ashleigh Horn, Amit Sil, Thomas J. Penfold, J. A. Gareth Williams, Valery N. Kozhevnikov, Fernando B. Dias

**Affiliations:** Department of Physics, Durham University South Road Durham DH1 3LE UK piotr.h.pander@durham.ac.uk; Department of Applied Sciences, Northumbria University Ellison Building Newcastle upon Tyne NE1 8ST UK valery.kozhevnikov@northumbria.ac.uk; Department of Chemistry, Durham University South Road Durham DH1 3LE UK j.a.g.williams@durham.ac.uk; Chemistry – School of Natural and Environmental Sciences, Newcastle University Newcastle upon Tyne NE1 7RU UK

## Abstract

A novel dinuclear platinum(ii) complex featuring a ditopic, bis-tetradentate ligand has been prepared. The ligand offers each metal ion a planar *O*^*N*^*C*^*N* coordination environment, with the two metal ions bound to the nitrogen atoms of a bridging pyrimidine unit. The complex is brightly luminescent in the red region of the spectrum with a photoluminescence quantum yield of 83% in deoxygenated methylcyclohexane solution at ambient temperature, and shows a remarkably short excited state lifetime of 2.1 μs. These properties are the result of an unusually high radiative rate constant of around 4 × 10^5^ s^−1^, a value which is comparable to that of the very best performing Ir(iii) complexes. This unusual behaviour is the result of efficient thermally activated reverse intersystem crossing, promoted by a small singlet–triplet energy difference of only 69 ± 3 meV. The complex was incorporated into solution-processed OLEDs achieving EQE_max_ = 7.4%. We believe this to be the first fully evidenced report of a Pt(ii) complex showing thermally activated delayed fluorescence (TADF) at room temperature, and indeed of a Pt(ii)-based delayed fluorescence emitter to be incorporated into an OLED.

## Abstract 2

Efficient thermally activated delayed fluorescence (TADF) in a brightly luminescent diplatinum(ii) complex results in significant enhancement of the radiative decay rate.

## Graphical abstract

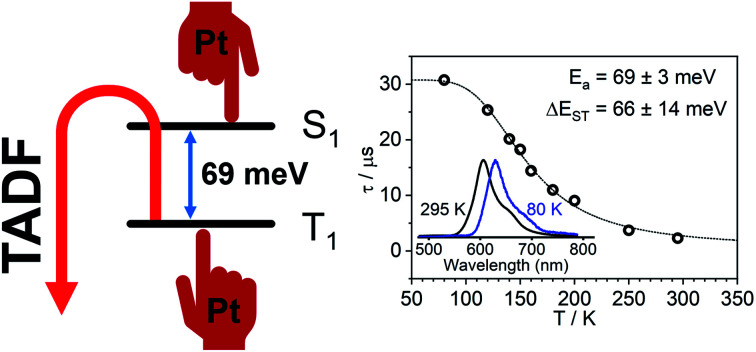

## Introduction

Organometallic Ir(iii) and Pt(ii) complexes have found important application in organic light-emitting diodes (OLEDs) as efficient emitters and triplet harvesting units that are capable of achieving internal quantum efficiencies up to 100%.^1–4^ The presence of the heavy metal in these complexes promotes stronger spin–orbit interactions and fast intersystem crossing resulting in up to 100% triplet formation. Moreover, their triplet radiative rate constants, *k*^T^_r_, are strongly increased by spin–orbit coupling induced by the metal. In the quest for deep red emitters, the most common strategies aimed at reducing the emission energy, in order to move into the red/NIR region, are based on destabilising the HOMO and stabilising the LUMO of the complex by using ligands that offer more extended conjugation. However, the associated reduction in metal character that necessarily accompanies such a strategy leads to lower *k*^T^_r_ and hence reduced quantum yields and longer excited state lifetimes. Long lifetimes may contribute to severe efficiency roll-off in OLED devices, because long-lived excitations are more likely to suffer from quenching caused by charge carriers.^1,5^ Ir(iii) complexes have generally been preferred in OLEDs over Pt(ii) analogues, since the near degeneracy of the filled metal d orbitals in a pseudo-octahedral d^6^ complex allows more efficient mixing of singlet character into the triplet than in a square-planar complex, thus yielding a higher radiative rate.^6^ In addition, deep-red and near infrared (NIR) complexes suffer from reduced photoluminescence quantum yields due to the well-known energy gap law: non-radiative decay through electronic-to-vibrational energy transfer is promoted in low-energy emitters.^7^

Consequently, there is a great motivation for molecular design strategies that lead to faster radiative rate constants in OLED emitters, such that emission can outcompete non-radiative decay. In this regard, a strategy of introducing a second metal centre is attracting increasing interest in this respect, as it appears to significantly enhance *k*^T^_r_ compared with mononuclear analogues.^8–12^ This approach has been found to reduce the energy difference (Δ*E*_ST_) between the lowest singlet and triplet excited states.^8,13^ A small Δ*E*_ST_ is important because it creates the possibility for triplet states to re-populate the singlet manifold, by using thermal energy to promote reverse intersystem crossing (RISC), in a similar way to that observed in compounds that show thermally activated delayed fluorescence (TADF).^14–16^ On the other hand a small Δ*E*_ST_ increases singlet–triplet mixing, enhancing the effect of spin–orbit coupling (SOC). TADF has been observed mostly in purely organic charge-transfer (CT) systems^14–18^ and in Cu(i) complexes,^19–21^ although there are an increasing number of examples with other metals too, including Ag(i)^22,23^ and Au(i),^24,25^ as well as a degree of delayed fluorescence in some Pd(ii) complexes (though phosphorescence remains the dominant mode of emission in that case).^26–29^

The reduction in Δ*E*_ST_ that has been found to accompany the change from mono to dinuclear complexes discussed above suggests that this bimetallic approach might allow the energy gap to be reduced to the extent that thermally activated RISC becomes efficient at room temperature. In this contribution, we describe a novel cyclometallated diplatinum(ii) complex of a bis-tetradentate, *O*^*N*^*C*^*N*–*N*^*C*^*N*^*O*-coordinating ligand, in which Δ*E*_ST_ is so small that efficient TADF occurs at room temperature. By exploiting the allowed S_1_ → S_0_ transition instead of the usual T_1_ → S_0_ route, the photoluminescence rate constant is significantly increased: *τ*_TADF_, the observed decay lifetime, is around 1 to 2 μs at room temperature. The observation of TADF opens up a pathway to achieve unprecedentedly short photoluminescence lifetimes in Pt(ii) complexes, competitive with those of the best Ir(iii) emitters.^10^

## Results and discussion

### Synthesis

**Scheme 1:**
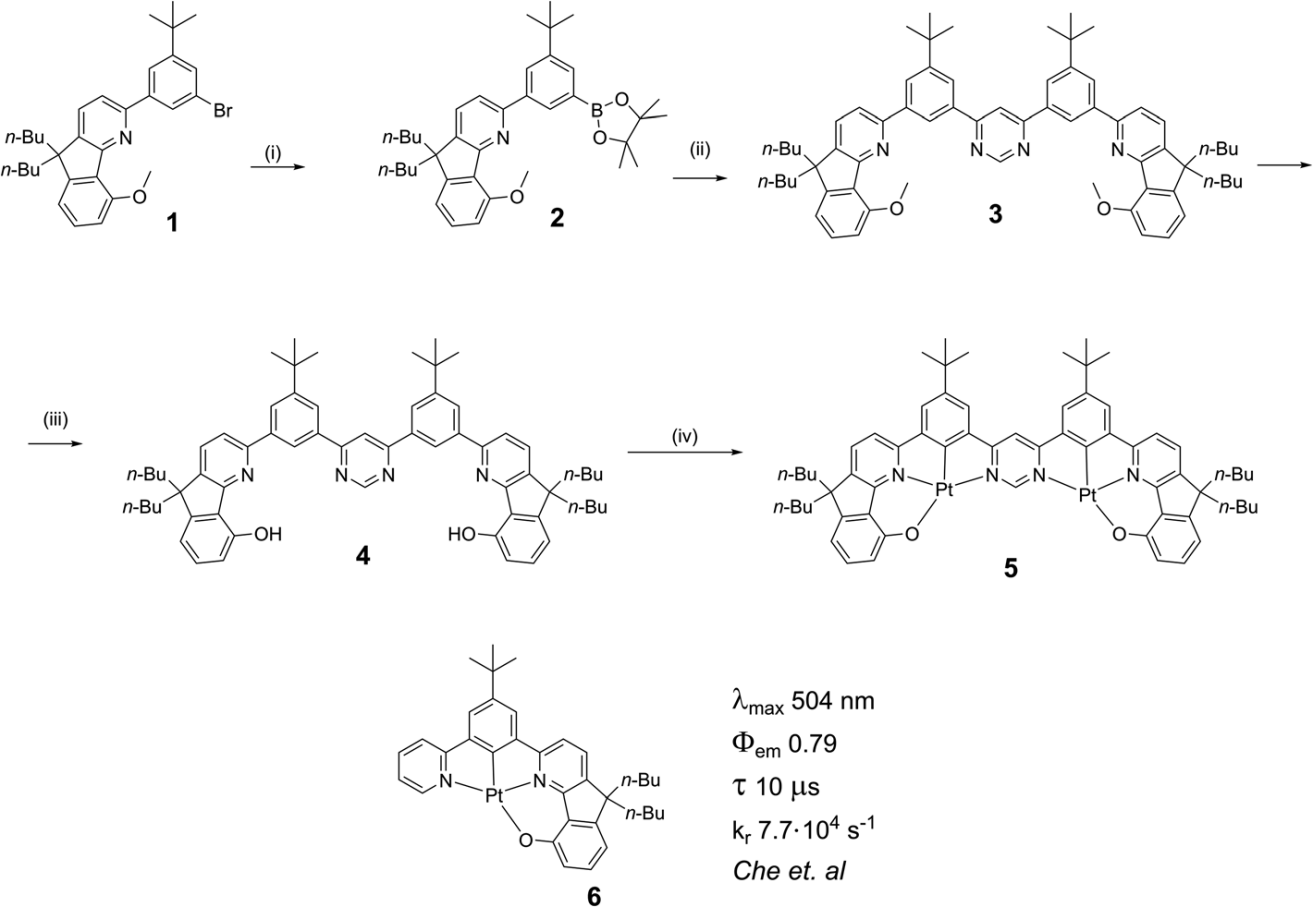
Synthesis of the ditopic, bis-tetradentate proligand **4** and its dinuclear Pt(ii) complex **5**. Reaction conditions: (i) bis(pinacolato)diboron, PdCl_2_(dppf), KOAc, 1,4-dioxane, 90 °C, 18 h, 87%; (ii) 4,6-dichloropyrimidine, Pd(PPh_3_)_4_, K_2_CO_3_, 1,4-dioxane/water, 85 °C, 24 h, 14%; (iii) pyridinium hydrochloride, 250 °C, 12 h, 70%; (iv) K_2_PtCl_4_, AcOH/CHCl_3_ 9 : 1, reflux, 3 d, 21%. The analogous mononuclear complex has been described by Che *et al.*‡‡The tabulated data and photoluminescence spectra shown in the ESI to the paper by Che *et al.*^32^ indicate a value of 504 nm for *λ*_max_ of **6** (in CH_2_Cl_2_), but a value of 537 nm is given in the table in the main text of the paper. The 537 nm figure should probably refer to the (0,1) vibrational shoulder, and we assume the 0,0 component to be at 504 nm.^32^

In our design of multimetallic emitters, we have drawn upon known highly luminescent monometallic building blocks and sought to fuse them together by means of a bridging heterocycle. Che and co-workers developed a series of intensely phosphorescent mononuclear Pt(ii) complexes with tetradentate *O*^*N*^*C*^*N*-coordinating ligands.^30,31^ We have adapted this structural motif to prepare dinuclear complexes with pyrimidine as a bridging heterocycle, to which both Pt(ii) centres are coordinated *via* the two nitrogen atoms (Scheme 1). The synthesis of the ditopic ligands starts with the known bromo derivative **1**,^30^ which was used to prepare the boronic acid derivative **2**. Pd-catalysed Suzuki cross-coupling of **2** with 4,6-dichloropyrimidine gave **3**, which was demethylated to give the target ditopic proligand **4**. The synthesis of the target dinuclear platinum(ii) complex **5** was carried out by reaction of proligand **4** with K_2_PtCl_4_ (>2 equiv.) in a mixture of acetic acid and chloroform (9 : 1 v/v) at reflux. The desired dinuclear complex was purified by column chromatography and characterised by ^1^H and ^13^C NMR spectroscopy and by high-resolution mass spectrometry.

**Fig. 1:**
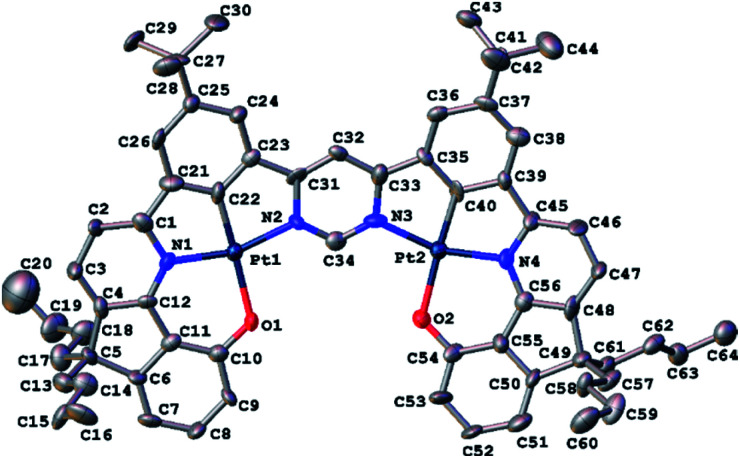
Molecular structure of **5** at 120 K. Hydrogen atoms are omitted for clarity; thermal ellipsoids are drawn at 50% probability. Metal ligating atom bond lengths (Å): Pt1–N1 1.988(1); Pt1–C22 1.846(1); Pt1–N2 2.016(1); Pt–O1 2.128(8); Pt2–N4 1.994(1); Pt2–C40 1.842(1); Pt2–N3 2.051(1); Pt2–O2 2.110(8).

Red crystals of **5** suitable for X-ray diffraction analysis were harvested by slow diffusion of methanol into a solution of the complex in dichloromethane. The molecular structure in the crystal is shown in Fig. 1; crystal structure data are summarized in Table S3.1 in the ESI.† The structure reveals the expected square-planar geometry around each metal ion, with only a small distortion from planarity across the whole molecule. The metal-ligating atom bond lengths are nearly identical for the two metal centres (see caption to Fig. 1). Within the crystal, the molecules pack with an off-centre head-to-tail arrangement between pairs of adjacent molecules (Fig. S3.1†). The shortest intermolecular Pt⋯Pt distance is 6.568(1) Å, showing that no Pt⋯Pt interactions occur in the ground state, whilst the shortest intermolecular distance of 4.21(1) Å between centres of parallel aromatic (pyrimidine) rings suggests that π–π interactions are minimal too (the corresponding shift of centroids is 2.61(2) Å).

### Theory

**Fig. 2:**
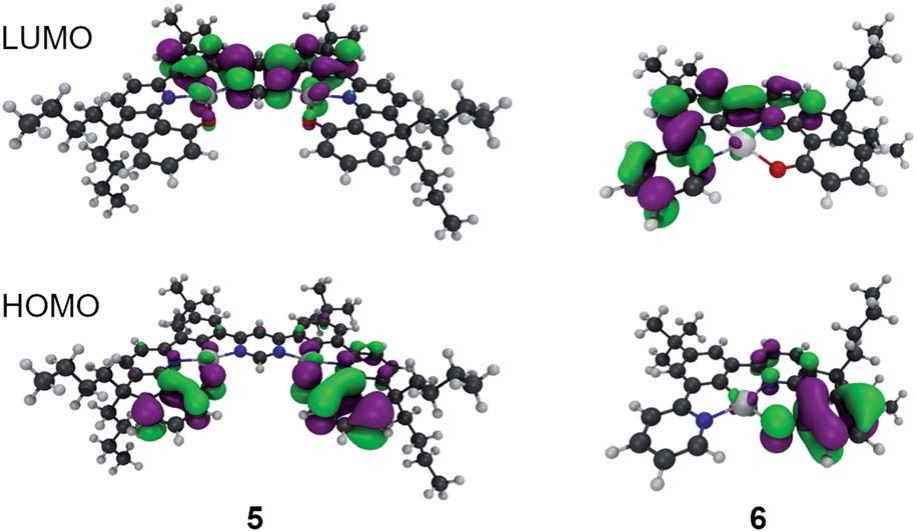
The LUMO (top) and HOMO (bottom) of complex **5** (left) and **6** (right).

For **6** the calculated phosphorescence wavelength is 537 nm (T_1_ → S_0_), in good agreement with the experimentally determined *λ*_max_ of emission of 504 nm.‡^32^ The HOMO and LUMO orbitals associated with this transition are shown in Fig. 2, indicating a charge-transfer state. On the other hand, for the bimetallic complex **5**, the calculated S_1_ → S_0_ fluorescence at 563 nm is quite close to the experimentally observed emission maximum of 612 nm. The HOMO and LUMO orbitals associated with this transition are shown in Fig. 2 and, like complex **6**, it exhibits charge-transfer character consistent with the solvatochromic shift observed experimentally. For both complexes the nature of the HOMO–LUMO transition is mostly intra-ligand charge transfer with admixtures of different Pt(ii) d orbitals. Importantly, the calculated S_0_ → S_1_ oscillator strength is 20-fold larger in complex **5** than in **6** (0.30 and 0.015 respectively) and Δ*E*_ST_ is substantially reduced from 0.37 to 0.18 eV on moving to the dinuclear complex. The reduction in Δ*E*_ST_ is associated with larger strength of charge transfer in **5** than in **6** and smaller HOMO–LUMO overlap in the former. Stronger electron accepting properties of pyrimidine (**5**) than pyridine (**6**) directly contribute to that. However, the S_1_–T_1_ spin–orbit coupling matrix element (SOCME) drops from 88 cm^−1^ for **6** to 10 cm^−1^ for **5** indicating that, despite the introduction of a second metal centre, the overall SOC is weaker in the dinuclear complex because the singlet and triplet states in **5** have much more similar characters than **6** and therefore the transition is more El-Sayed forbidden.^33^

### Photophysics in solution

#### (i) Steady-state UV-visible absorption and photoluminescence

**Fig. 3:**
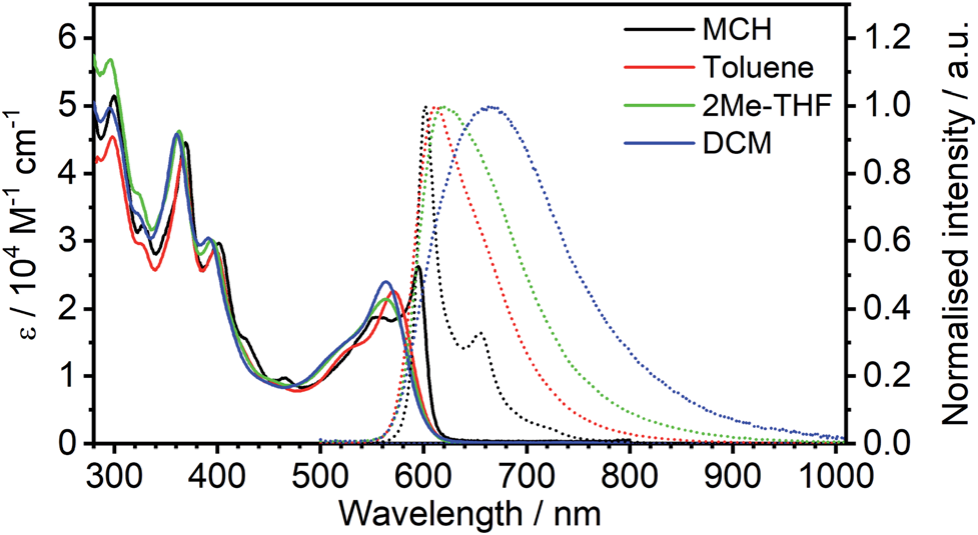
Absorption and normalised photoluminescence spectra of **5** in various solvents at room temperature.

The absorption spectra of complex **5** recorded in four solvents of different polarity are shown in Fig. 3. Compared to simple cyclometallated Pt(ii) complexes, the most striking features are the long wavelength (around 600 nm) and remarkably high intensity (*ε* > 20 000 M^−1^ cm^−1^) of the lowest energy absorption bands, properties which are in common with related di-Pt(ii) complexes previously reported.^8,11,12^ This band shows significant negative solvatochromism, blue-shifting by 32 nm (110 meV, 960 cm^−1^) in DCM with respect to methylcyclohexane (MCH) (Table 1). There is also a trend to broadening of the absorption bands and poorer resolution of vibrational structure as the solvent polarity increases.

**Table 1:** Summary of absorption and photoluminescence properties of **5** in degassed solvents at room temperature

Solvent	*λ*_abs_ a/nm (*ε*/10^3^ M^−1^ cm^−1^)	*λ*_em_ b/nm	Stokes shift^c^ nm/cm^−1^/meV	*Φ*_PL_ d	*τ* e/μs	*k* ^TADF^_r_ f/10^5^ s^−1^	*k*_nr_ g/10^5^ s^−1^
MCH	595 (26), 555 (19), 465 (9.8), 401 (30), 369 (45), 327 (32), 300 (52)	602, 654	7/195/24	0.83 ± 0.09	2.1 ± 0.1	4.0 ± 0.6	0.8 ± 0.5
Toluene	571 (23), 524sh (14), 459 (8.8), 398 (29), 366 (42), 327sh (29), 298 (45)	612	41/1173/145	0.68 ± 0.08	1.2 ± 0.1	5 ± 1	2.6 ± 0.9
2Me-THF	564 (21), 517sh (14), 394 (30), 362 (46), 323sh (37), 295 (57)	618	54/1549/192	0.04 ± 0.01	0.11 ± 0.01	4 ± 2	92 ± 9
DCM	563 (24), 512sh (13), 392 (30), 361 (46), 324 (34), 296 (50)	664	101/2702/335	0.02 ± 0.01	0.055 ± 0.002	4 ± 2	180 ± 9

a Main absorption maxima and associated absorption coefficients.

b Emission maxima.

c The ‘apparent’ Stokes shift; *i.e.*, the difference between *λ*_abs_ (for the lowest-energy band) and *λ*_em_.

d Photoluminescence quantum yield recorded using tetraphenylporphyrin in air-equilibrated acetonitrile as the standard, *Φ*_PL_ = 0.075.^34,35^

e Photoluminescence lifetime.

f TADF radiative rate constant calculated assuming that the emissive state is formed with unit efficiency: *k*^TADF^_r_ = *Φ*_PL_/*τ*.

g Effective non-radiative rate constant, *k*_nr_ = (1 − *Φ*_PL_)/*τ*.

In MCH, the complex is highly emissive, with *λ*_max_ = 602 nm. The form of the spectrum resembles the mirror image of the absorption spectrum and the Stokes shift of only 7 nm (24 meV, 195 cm^−1^) is extraordinarily small for a 3^rd^-row transition metal complex. Such complexes normally emit by phosphorescence from the T_1_ state, as opposed to fluorescence from the S_1_. In that case, a mirror-image relationship with the absorption spectrum is not to be expected, since the emitting state is different from the spin-allowed states to which absorption occurs. Thus, the low-energy absorption band may be associated with the S_0_ → S_1_ transition and related photoluminescence to the S_1_ → S_0_ transition. Moreover, the apparent Stokes shift in such complexes will comprise not only the stabilisation of the emissive excited state by reorganisation prior to emission (as occurs in almost all fluorophores) but also Δ*E*_ST_. Such a small Stokes shift not only means that there is very little structural reorganisation in either the excited (S_1_ or T_1_) state, but also that there must be a very small Δ*E*_ST_, if indeed the emission is emanating from a triplet state. The highly rigid nature of the choromophore might reasonably account for the first point, with the rigidity being reinforced by the bound metal ions (see Fig. 1): it is interesting to note that the apparent Stokes shift is smaller even than that of most typical fluorescent molecules emitting *via*^1^π–π* excited states.^36,37^ A negligibly small Δ*E*_ST_, on the other hand, seems to make direct triplet decay T_1_ → S_0_ implausible due to the competition with reverse intersystem crossing (RISC). On the contrary, taken together, these observations imply that the emissive state is actually the singlet S_1_ at room temperature, as opposed to T_1_, and potentially also that rapid RISC competes with T_1_ → S_0_ phosphorescence.^38^

The emission shows positive solvatochromism, with the maximum red-shifting by 62 nm (190 meV, 1550 cm^−1^) in DCM with respect to MCH and intermediate values in toluene and 2-methyltetrahydrofuran (2MeTHF) (Fig. 3). Again, the spectra are seen to become less vibrationally resolved with increasing polarity. Such changes are typical experimental signs of charge-transfer character in the emissive state.^39,40^ Perhaps the most striking observation from steady-state measurements, however, is the collapse in the photoluminescence quantum yield (PLQY) upon increasing polarity: the PLQY falls from 0.83 in MCH (an unusually high value for a red-emitting Pt(ii) complex) to only 0.02 in DCM (Table 1). It is useful to note at this point that the mononuclear analogue of **5**, namely **6**, displays negative solvatochromism in absorption, but no solvatochromism was reported in its phosphorescence.^32^ Such behaviour might be interpreted in terms of the S_1_ state of the **6** being of CT character, but not the T_1_. This finding is informative in understanding the nature of the excited states in complex **5**, discussed further in Section 5c in the ESI.†

#### (ii) Time-resolved photoluminescence

**Fig. 4:**
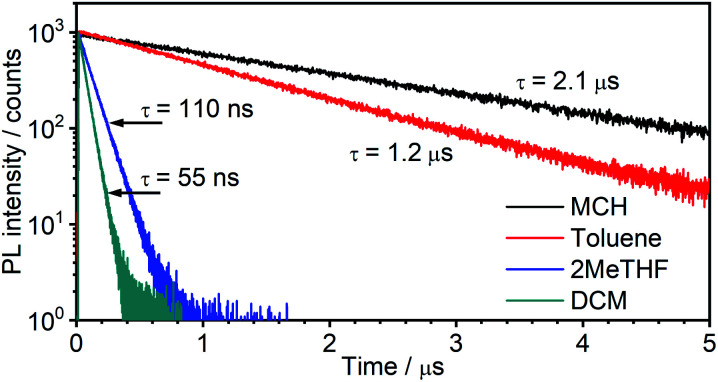
Photoluminescence decay of **5** in the four solvents colour-coded as in Fig. 3.

The time-resolved photoluminescence decay traces of **5** are shown in Fig. 4; the lifetimes and the calculated radiative and non-radiative rate constants are compiled in Table 1. The emission decays follow single exponential kinetics in each case. Based on the lifetimes and quantum yields, and assuming the emitting state is formed with unit efficiency, the radiative rate constant *k*^TADF^_r_ is calculated to be around 4 × 10^5^ s^−1^. Such a value is typical of metal complexes with very strong SOC, involving predominantly ^3^MLCT excited states with large d orbital admixture from the metal, and is normally confined to iridium(iii)-based emitters {*e.g.*, for the archetypal *fac*-Ir(ppy)_3_, *k*_r_ is around 5 × 10^5^ s^−1^}.^41^ Values for mononuclear Pt(ii) complexes with comparable quantum yields are lower, of the order of 10^4^–10^5^ s^−1^, resulting in longer lifetimes;^10,32,42,43^ for example, *k*_r_ for the monometallic analogue of **5** is nearly an order of magnitude lower, at 7.7 × 10^4^ s^−1^.^32^

Such a high rate of emission from a triplet state would require a strong d orbital admixture and an unequivocal ^3^MLCT formulation to the emissive state. Strikingly, this is not the case in **5**: the calculations (Theory, above) showed that the emissive state is predominantly of ^3^LC character, with only a small d_Pt_ admixture. These observations support the tentative hypothesis from the steady-state data that **5** emits from the S_1_ state at room temperature in MCH. In this scenario, the S_1_ and T_1_ states remain in a fast equilibrium that is governed by RISC and ISC. The rates of these two processes are likely to be several orders of magnitude higher than in metal-free TADF emitters, >10^12^ s^−1^ for ISC (most crucially *k*_ISC_ ≫ *k*^S^_r_), due to the strong SOC induced by the metal, and much higher than the delayed fluorescence radiative rate constant. Changes in the S_1_–T_1_ equilibrium constant are governed by the Boltzmann equation.^43,44^

In terms of the solvent dependence, the data (Table 1) show that there is a significant rise of *k*^TADF^_nr_ with solvent polarity, but *k*^TADF^_r_ stays virtually unchanged. The latter finding suggests the TADF mechanism to remain at work not only in low – but also higher polarity solvents. The increase in *k*_nr_ is much larger than what would be expected based just on the increased vibrational quenching as the emissive state falls in energy (*e.g.*, the energy is only slightly lower in 2MeTHF than toluene, but *k*^TADF^_nr_ increases by more than an order of magnitude). Solvation effects may be at work that influence the relative energies of the S_1_, T_1_ and higher-lying states. Interestingly, a similar observation has been made in a Pt(0) complex that displays TADF, namely Pt(*P*^*P*-binap)_2_, where it was attributed to the solvation effect of a higher-lying excited state involved in the non-radiative process.^45^ The lifetimes both of the new complex **5** and of Pt(*P*^*P*-binap)_2_ follow the same function of solvent polarity (Fig. S5.4†), indicating a clear correlation of these two parameters.

### Photophysics in amorphous polymeric matrices

**Fig. 5:**
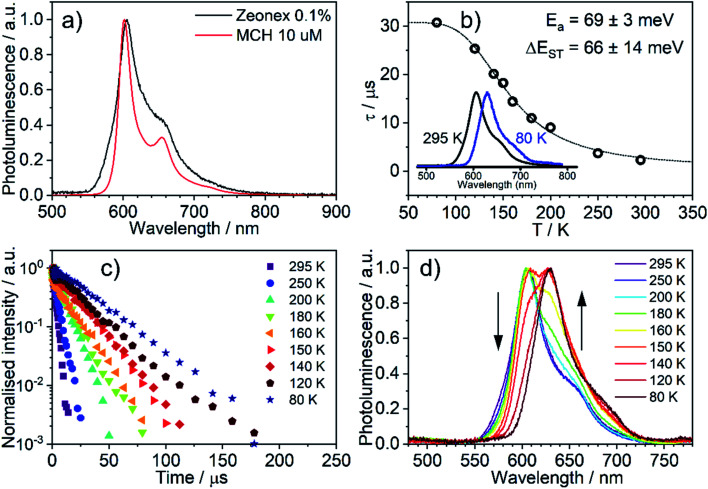
Photoluminescence of **5** in Zeonex® (0.1% w/w): (a) photoluminescence spectra in MCH and Zeonex® thin film; (b) temperature dependence of decay lifetime; (c) photoluminescence decay traces at temperatures from 295 to 80 K; (d) photoluminescence spectra at temperatures from 295 to 80 K.

The emission of **5** in Zeonex® thin films§§Transparent solid films offer a useful medium for studying emissive molecules over a wide range of temperatures, without presenting issues encountered in solvents, such as expansion, contraction, change in oxygen concentration, or diffusion with temperature. The rigid environment prohibits large structural reorganisation of the emitter in the excited state. Zeonex® is an amorphous cyclo-olefin polymer^54^ which has been widely used to study TADF materials.^40,55–57^ (Fig. 5a) resembles that in MCH. Both are non-polar aliphatic media, but the spectrum in the film appears slightly broader. The photoluminescence decays in Zeonex® (Fig. 5b and c) show a clear temperature dependence, with lifetime increasing as temperature decreases, but without the associated increase of photoluminescence intensity (Fig. S5.5†), in agreement with known models for thermally activated delayed fluorescence (TADF).^43,44^ The temperature dependence of the emission profile can be interpreted in terms of dominant higher-energy fluorescence at temperatures in excess of about 200 K, and lower-energy phosphorescence at lower temperatures (Fig. 5d). These observations are consistent with emission being dominated by TADF at room temperature.^43,46,47^ Importantly, with the emergence of RISC and thus TADF, the effective photoluminescence decay rate of **5***k*^TADF^_r_ = 4 × 10^5^ s^−1^ at room temperature is much higher than that of its mononuclear analogue **6**, *k*^T^_r_ = 7.7 × 10^4^ s^−1^, despite the significantly larger SOC calculated for the latter.

**Fig. 6:**
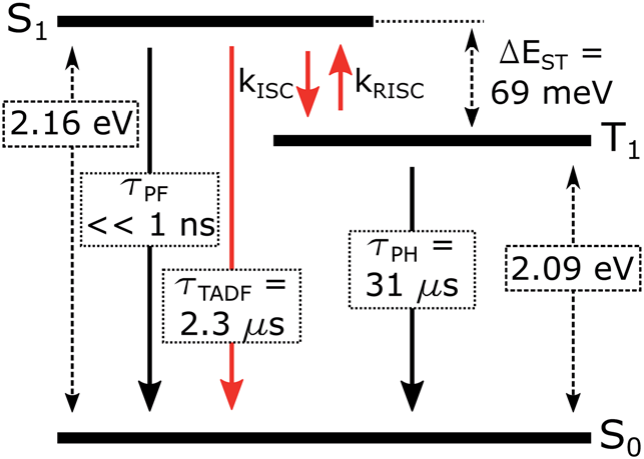
Excited state diagram of **5** in Zeonex®.

When Δ*E*_ST_ is small, *i.e.*, below 0.2–0.4 eV, the T_1_ state can be deactivated *via* RISC (T_1_ → S_1_) followed by delayed fluorescence (S_1_ → S_0_), in addition to usual radiative (phosphorescence, *k*_PH_ = *τ*_PH_^−1^) and non-radiative Σ*k*_nr_ processes (Fig. 6). At higher temperatures the equilibrium concentration of S_1_ states is sufficient to allow for fast radiative decay through the singlet manifold, *k*^TADF^_r_ ≫ *k*_PH_. However, *k*^TADF^_r_ decreases with temperature, due to the equilibrium shifting towards the T_1_. At temperatures close to 80 K, *k*^TADF^_r_ ≪ *k*_PH_ so that only phosphorescence is observed.

It should be mentioned that the emission spectrum at a given temperature always remains a superposition of two different spectra, indicating the existence of two distinct emissive states: delayed fluorescence from S_1_ and phosphorescence from T_1_. Such a composite emission profile is invariant for any delay time at a specific temperature, indicating that the two states remain in equilibrium and decay with identical lifetimes. This is perceived as a fast singlet–triplet cycling process as described earlier in the literature^43,44,48^ and confirms the assumptions of the model and eqn (1).

The prompt fluorescence of organometallic TADF emitters, such as Cu(i) complexes,^19,43^ is very short-lived and often cannot be directly recorded. Previous studies have shown the prompt fluorescence of several complexes to be in a range of femto- to picoseconds^45,49,50^ which exceeds the time resolution of the equipment used in this work (≈1–2 ns). Therefore, the lack of visible prompt fluorescence in the photoluminescence decay of **5** is not surprising. However, despite the lack of direct observation of prompt fluorescence, its radiative rate constant can still be estimated indirectly using eqn (1).^43,44^1
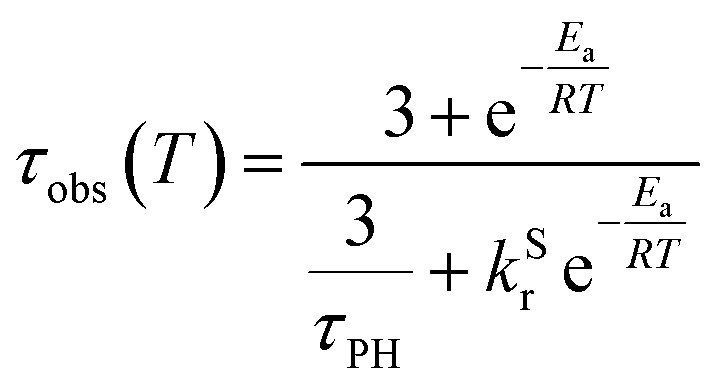


The variation of the photoluminescence lifetime with temperature from 2.3 ± 0.1 μs at 295 K up to 30.7 ± 0.8 μs at 80 K can be described by eqn (1), where *τ*_obs_(*T*) is the observed emission lifetime (s); *E*_a_ is the activation energy of the reverse intersystem crossing process in J mol^−1^; *τ*_PH_ is the phosphorescence lifetime (s); *k*^S^_r_ is the radiative rate constant of singlet state (s^−1^); *R* is the universal gas constant, 8.314 J mol^−1^ K^−1^; and *T* is the sample temperature in K. The observed lifetime value at 80 K is assigned as the closest approximation to the phosphorescence lifetime, *τ*_PH_ in eqn (1). This is justified by the phosphorescent-only origin of the emission at 80 K (see Fig. 5d). By fitting the emission lifetime as a function of *T*, *τ*_obs_(*T*), using eqn (1), an activation energy *E*_a_ = 69 ± 3 meV is determined. This value is in perfect agreement with the singlet–triplet energy difference Δ*E*_ST_ = 66 ± 14 meV determined from the onset wavelengths of the fluorescence and phosphorescence spectra. Such good agreement confirms that thermally activated RISC is governing TADF between the T_1_ and S_1_ states, with an energy barrier *E*_a_ = Δ*E*_ST_. Furthermore, the radiative rate constant of prompt fluorescence can also be obtained from the fit, *k*^S^_r_ = (1.5 ± 0.3) × 10^7^ s^−1^. However, this rate constant is close to that usually observed in typical metal-free intra-/intermolecular CT TADF molecules.^51^ These results are clearly consistent with the solution properties and a clear correspondence can be made between the photophysical properties in thin film and in a solution. The photophysical behaviour in polystyrene is also very similar to that in Zeonex® (Fig. S5.6–S5.10†), where the same experiments gave *E*_a_ = 68 ± 4 meV and *k*^S^_r_ = (1.3 ± 0.4) × 10^7^ s^−1^.

### OLED devices

Emitters with short excited state lifetimes, such as in complex **5**, are highly desirable for OLEDs. However, there are other issues that need to be addressed to obtain devices with excellent performance; for example, for solution processed devices, solubility is of paramount importance. Most organometallic OLED emitters are insoluble in non-chlorinated solvents or only weakly soluble and thus can only be vacuum-deposited,^52,53^ Complex **5**, however, is readily soluble in toluene and therefore perfectly suited for solution-processed devices. It was found earlier^8^ that TPD : PBD (60 : 40, w/w) blend {TPD – *N*,*N*′-bis(3-methylphenyl)-*N*,*N*′-bis(phenyl)-benzidine; PBD – 2-(4-biphenyl)-5-(4-*tert*-butylphenyl)-1,3,4-oxadiazole} is well-suited as a host for red-emitting bimetallic Pt(ii) complexes.

Therefore, the optimum **5** prototype device consisted of a hole injection layer (HIL 1.3N, 45 nm) and a hole transport/electron blocking layer (PVKH – poly(9-vinylcarbazole), *M*_w_ = 1.1 × 10^6^ to 10 nm). PVKH was spun onto the annealed HIL 1.3N layer from a chloroform : chlorobenzene (95 : 5 v/v) solution. These were followed by the emitting layer spun from toluene solution, while all the other layers were thermally deposited. As no hole-blocking layer was necessary, the whole device architecture consisted of: ITO|HIL 1.3N (45 nm)|PVKH (10 nm)|TPD : PBD (60 : 40 w/w) co 5% **5** (30 nm)|TPBi (50 nm)|LiF (0.8 nm)|Al (100 nm). TPBi {1,3,5-tris(1-phenyl-1*H*-benzimidazol-2-yl)benzene} plays a role of the electron transport layer. The superior solubility of **5** in non-chlorinated solvents allows for the use of PVKH as an electron blocking layer.

**Fig. 7:**
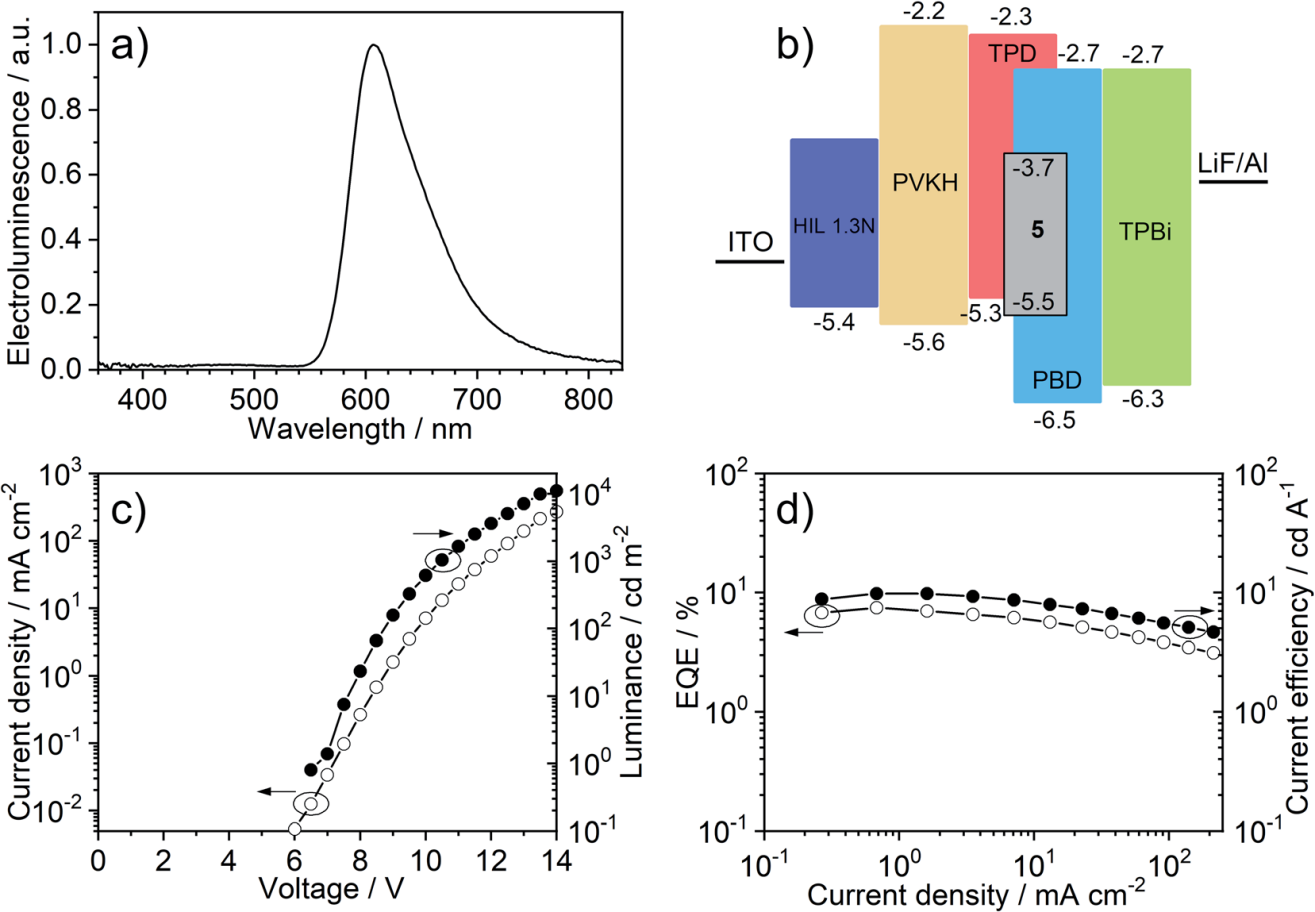
OLED device characteristics: (a) electroluminescence spectrum; (b) OLED device architecture; (c) current–voltage and luminance; (d) external quantum efficiency (EQE) *vs.* current efficiency.

The OLED device (Fig. 7) shows red emission, CIE (0.62, 0.37), *λ*_EL_ = 607 nm and full width at half maximum, FWHM = 75 nm. The electroluminescence spectra in the OLED host are broadened with respect to solution and polymer matrix. This effect is not due to the interaction of **5** with the TPD : PBD blend *per se* (*i.e.*, stabilisation of the emitter CT state in the host) but due to dispersion of emissive state energy in the blend (see detailed discussion in Section 5c of the ESI†). The EQE reaches a maximum of 7.4% which is fully consistent with the luminescence yield in film *Φ*_PL_ = 0.31 ± 0.05. In this case, with an out-coupling factor of 0.2–0.3 in a flat device fabricated on a glass substrate, the maximum EQE can reach up to ≈6–9%. The low *Φ*_PL_ in film, compared with solution, is probably a result of the molecule forming non-emissive aggregates in the solid film (see Sections 5a and 5c of the ESI†). This could be related to the molecule's planar structure that promotes molecular stacking and may be improved in the future by modification of the molecular design. The OLED shows a turn-on voltage *V*_ON_ = 7 V at 1 cd m^−2^ and a maximum luminance of 11 000 cd m^−2^ while its maximum radiosity reaches 15.1 mW cm^−2^. Red-electroluminescent devices based on host TPD : PBD and using a PVKH electron blocking layer achieve large current densities while the short-lived photoluminescence of **5** ensures that efficiency roll-off is minimised.

## Conclusions

This work presents the first fully evidenced dinuclear platinum(ii) TADF emitter and the first organoplatinum compound used in TADF OLEDs. The remarkable and unique properties of this complex open a pathway to the design of a new family of luminescent compounds – TADF Pt(ii) complexes. This new approach to the bimetallic strategy allows for a remarkably small singlet–triplet gap of 66 ± 14 meV and short ∼1–2 μs photoluminescence lifetimes. The design of this emitter not only leads to efficient luminescence but also to superior solubility, facilitating fabrication of solution-processed OLED devices.

The use of a rigid di-Pt(ii) TADF complex **5** brings significant improvements in comparison with other emitters, such as a large radiative rate constant, *k*^TADF^_r_ = 4 × 10^5^ s^−1^ and small FWHM = 75 nm in OLED host and remarkable 22 nm in methylcyclohexane. These features would render an optimised family of derivatives of **5** of potential interest, for example, in eliminating contamination by visible red emission in NIR OLEDs.

## Author contributions

P. P. – conceptualization, formal analysis, investigation, visualization, writing – original draft, writing – review & editing; R. D. – investigation; A. V. Z. – investigation; A. H. – investigation; A. S. – investigation; T. J. P. – investigation, resources, validation, writing – review & editing; J. A. G. W. – conceptualization, funding acquisition, project administration, supervision, writing – review & editing; V. N. K. – conceptualization, funding acquisition, project administration, writing – original draft, writing – review & editing; F. B. D. – funding acquisition, project administration, resources, validation, writing – original draft, writing – review & editing.

## Conflicts of interest

There are no conflicts to declare.

## Supplementary Material

SC-012-D1SC00160D-s001

SC-012-D1SC00160D-s002

SC-012-D1SC00160D-s003

SC-012-D1SC00160D-s004

SC-012-D1SC00160D-s005

SC-012-D1SC00160D-s006
