# Supplementary material for: Exceptionally fast radiative decay of a dinuclear platinum complex through thermally activated delayed fluorescence
Source: Chem Sci. 2021 Mar 22;12(17):6172–80. doi: 10.1039/d1sc00160d (PMC8098751; doi:10.1039/d1sc00160d)
Supplement: SC-012-D1SC00160D-s005 [file SC-012-D1SC00160D-s005.pdf]

Electronic Supplementary Material (ESI) for Chemical Science.
This journal is © The Royal Society of Chemistry 2021

Supporting information

Exceptionally Fast Radiative Decay of a Dinuclear Platinum Complex Through Thermally Activated Delayed Fluorescence

Piotr Pander, Ruth Daniels, Andrey V. Zaytsev, Ashleigh Horn, Amit Sil,
Thomas J. Penfold, J. A. Gareth Williams, Valery N. Kozhevnikov
and Fernando B. Dias

Table of contents

1. General 2
2. Synthesis 5
3. X-ray diffraction analysis 10
4. Theory 12
5. Photophysics 13
   a) Solution state 13
   b) Solid film (polymer matrix) 15
   c) Studies in an OLED host material 18
6. Electrochemistry 22
7. OLED devices 23
8. References 24

1. General

Theory

To assist the interpretation of the experimental results, we have performed density functional theory (DFT) and time-dependent density functional theory (TD-DFT) simulations on the di- and mono-nuclear complexes **5** and **6**, respectively using the ORCA quantum chemistry software[1]. All simulations were performed within the approximation of the LC-BLYP (ω=0.13 a_0^{-1}) exchange and correlation functional.[2,3] A def2-SVP basis set was used for all atoms except Pt, for which a def2-ECP basis was used. Spin-orbit coupling (SOC) calculations were performed using the Amsterdam Density Functional (ADF) code.[4–6] The SOC matrix elements (SOCME) were computed with the perturbative approach developed by Ziegler and Wang.[7] A DZP basis set was used for all atoms except Pt, for which a TZP basis set was used. Scalar relativistic effects were accounted for using a zeroth-order relativistic approximation (ZORA).[8,9]

Electrochemistry

Cyclic voltammetry was conducted in a three-electrode, one-compartment cell. All measurements were performed using 0.1 M Bu_4NBF_4 (99%, Sigma Aldrich, dried) solution in dichloromethane (ExtraDry AcroSeal®, Acros Organics). Solutions were nitrogen-purged prior to measurement and the measurement was conducted in a nitrogen atmosphere. Electrodes: working (Pt disc d = 1 mm), counter (Pt wire), reference (Ag/AgCl calibrated against ferrocene). All cyclic voltammetry measurements were performed at room temperature with a scan rate of 50 mV s^{-1}.

The ionization potential (IP) and electron affinity (EA) are obtained from onset redox potentials; these figures correspond to HOMO and LUMO values, respectively. The ionization potential is calculated from onset oxidation potential $IP = E_{ox}^{CV} + 5.1$ and the electron affinity is calculated from onset reduction potential $EA = E_{red}^{CV} + 5.1$.[10,11,12,13] An uncertainty of ±0.02 V is estimated for the electrochemical onset potentials.

Photophysics

Absorption spectra of 10^{-6} – 10^{-5} M solutions were recorded with UV-3600 double beam spectrophotometer (Shimadzu). Photoluminescence (PL) spectra of solutions and films were

recorded using a QePro compact spectrometer (Ocean Optics) or FluoroLog fluorescence spectrometer (Jobin Yvon). Phosphorescence decays were recorded using nanosecond gated luminescence and lifetime measurements (from 400 ps to 1 s) using the third harmonic of a high-energy pulsed Nd:YAG laser emitting at 355 nm (EKSPLA). The emitted light was focused onto a spectrograph and detected with a sensitive gated iCCD camera (Stanford Computer Optics) having sub-nanosecond resolution. Time-resolved measurements were performed by exponentially increasing gate and integration times. Further details are available in reference [14]. Time-resolved decays in solution were recorded with a Horiba DeltaFlex TCSPC system using a 330 nm SpectraLED light source. Temperature-dependent experiments were conducted using a liquid nitrogen cryostat VNF-100 (sample in flowing vapour, Janis Research) under nitrogen atmosphere, while measurements at room temperature were recorded under vacuum in the same cryostat. Solutions were degassed using five freeze-pump-thaw cycles. Thin films in Zeonex® and OLED host matrix were obtained from toluene solution while polystyrene solid films were deposited from chloroform. The films were fabricated through spin-coating and dried under vacuum at room temperature. Solid state emission spectra and photoluminescence quantum yield were obtained using an integrating sphere (Labsphere) coupled with a 365 nm LED light source and QePro (Ocean Optics) detector.

OLED devices

OLEDs were fabricated by spin-coating / evaporation hybrid method. The hole injection layer (Heraeus Clevios HIL 1.3N), electron blocking/hole transport layer (PVKH), and emitting layer (TPD:PBD + dopant) were spin-coated, whereas the electron transport layer (TPBi) and cathode (LiF/Al) were evaporated. Devices of 4 × 2mm pixel size were fabricated. TPD – *N*,*N*' -Bis(3-methylphenyl)-*N*,*N*' -bis(phenyl)-benzidine (LUMTEC), PVKH – poly(9-vinylcarbazole) (MW = 1 100 000, Sigma Aldrich), PBD - 2-(biphenyl-4-yl)-5-(4-*tert*-butylphenyl)-1,3,4-oxadiazole (99%, Sigma Aldrich), PBD - 2-(biphenyl-4-yl)-5-(4-*tert*-butylphenyl)-1,3,4-oxadiazole (99%, Sigma Aldrich), TPBi - 2,2',2''-(1,3,5-Benzinetriyl)-tris(1-phenyl-1-*H*-benzimidazole) (sublimed, LUMTEC), LiF (99.995%, Sigma Aldrich), and Aluminium wire (99.9995%, Alfa Aesar) were purchased from the companies indicated in parentheses. OLED devices were fabricated using pre-cleaned with ozone plasma indium-tin-oxide (ITO) coated glass substrates with a sheet resistance of 20 Ω cm^{-2} and ITO thickness of 100 nm. Heraeus Clevios HIL 1.3N was spun-coated and annealed onto a hotplate at 200 ˚C for 3 min to give a 45 nm film. Electron blocking/hole transport layer (PVKH), was spun from chloroform:chlorobenzene (95:5 v/v) (3

mg/mL) and annealed at 50 ˚C for 5 min to give a 10 nm film. Emitting layer was spun from toluene solution of TPD:PBD (60:40 w/w) with total concentration of host 10 mg/mL. The dopant was dissolved in the solution of blend host in order to obtain final 5% concentration of the emitting layer. The solution was spun onto the PVKH layer and then annealed at 50 ˚C for 5 min giving 30 nm film. All solutions were filtrated directly before application using a PVDF or PTFE syringe filter with 0.45 μm pore size. All other electron transport and cathode layers were thermally evaporated using Kurt J. Lesker Spectros II deposition system at 10^{-6} mbar. All organic materials and aluminium were deposited at a rate of 1 Å s^{-1}. The LiF layer was deposited at a rate of 0.1–0.2 Å s^{-1}. Characterisation of OLED devices was conducted in 10 inch integrating sphere (Labsphere) connected to a Source Measure Unit and coupled with a spectrometer USB4000 (Ocean Optics). Further details are available in reference [15].

2. Synthesis

Compound **1** was synthesized as described previously.[16]

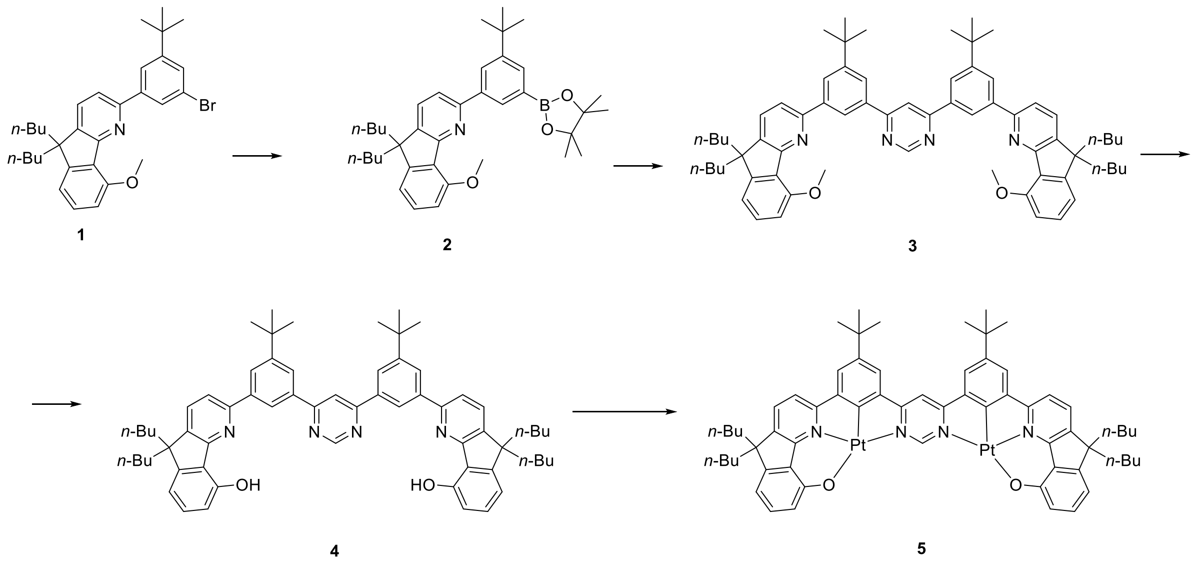

Boronic acid derivative **2.**

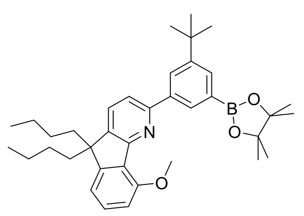

The bromo derivative **1** (1.26 g, 2.42 mmol) was added to a dry round-bottomed flask flushed with argon. Anhydrous 1,4-dioxane (50 mL) was added. The reaction mixture was stirred. Bis(pinacolato)diboron (0.920 g, 3.62 mmol) and potassium acetate (0.494 g, 5.03 mmol) were added and the reaction mixture was deairated by bubbling argon through the mixture for 15 min. [1,1′-Bis(diphenylphosphino)ferrocene]dichloropalladium(II) (0.0986 g, 0.121 mmol) was added and the reaction mixture was further deairated for 10 min. The reaction mixture was stirred at 90°C for 18 h. The reaction mixture was allowed to cool to room temperature, diluted with water (50 mL) and extracted with diethyl ether (3X 50 mL). The organic phase was washed with water (2X30 mL), dried with $MgSO_4$ and filtered. The solvent was evaporated to dryness under reduced pressure. The product was purified by column chromatography (silica gel, gradient elution with hexane:ethyl acetate 100:0 to 100:10) to give compound **2** as a brown oil. Yield: 1.20 g, 2.11 mmol, 87%

^{1}H NMR (400 MHz, $CDCl_3$): δ 8.65 (t, 1H, *J* = 2.1), 8.30 (s, 1H), 7.87 (m, 1H), 7.78 (d, 1H, *J* = 7.8), 7.62 (d, 1H, *J* = 7.8), 7.38 (t, 1H, *J* = 7.8), 7.00 (d, 1H, *J* = 6.9), 6.92 (d, 1H, *J* = 8.2), 4.11 (s, 3H), 2.01-1.96 (m, 4H), 1.46 (s, 9H), 1.38 (s, 12H), 1.06 (sext, 4H, *J* = 22.0, 14.8, 7.3), 0.66-0.55 (m, 10H).

^{13}C DEPT135 NMR (100 MHz, $CDCl_3$): δ 171.17 (C), 160.26 (C), 156.22 (C), 156.03 (C), 154.06 (C), 150.65 (C), 142.01 (C), 138.58 (C), 131.95 (CH), 130.27 (CH), 130.18 (CH), 130.11 (CH), 128.28 (C), 127.00 (CH), 116.98 (CH), 114.94 (CH), 109.48 (CH), 55.87 (CH_3),

53.00 (C), 40.03 (CH_2), 34.94 (C), 31.45 ($(CH_3)_3$), 26.07 (CH_2), 24.95 (CH_3), 23.06 (CH_2), 21.07 (C), 13.84 (CH_3).

Compound ***3***

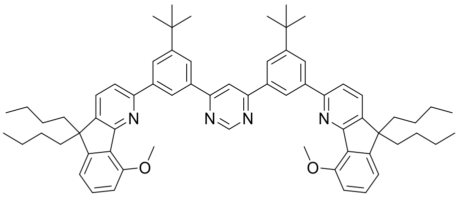

The boronic derivative **2** (4.38 g, 7.72 mmol) was added to a dry round-bottomed flask flushed with argon. Anhydrous 1,4-dioxane (100 mL) was added and the reaction mixture was stirred. 4,6-Dichloropyrimidine (0.460 g, 3.09 mmol) and 2M K_2CO_3 solution (2.56 g, 18.5 mmol, 9.50 mL) were added and the reaction mixture was degassed under argon for 10 min. Tetrakis(triphenylphosphine)palladium(0) (0.214 g, 0.185 mmol) was added and the reaction mixture degassed for a further 10 min. The reaction mixture was heated at 95°C for 24 h. The reaction mixture was cooled to room temperature and extracted with ethyl acetate. The organic phase was washed with water, separated and dried with $MgSO_4$. The solvent was evaporated to dryness. The crude product was purified by column chromatography (silca gel, gradient elution with hexane:ethyl acetate) to give the product as a pale yellow oil

Yield: 0.400 g, 0.417 mmol, 14%

^{1}H NMR (400 MHz, $CDCl_3$): δ 9.43 (d, 1H, *J* = 0.9), 8.69 (s, 2H), 8.61 (t, 2H, *J* = 1.6), 8.38 (d, 1H, *J* = 0.9), 8.32 (t, 2H, *J* = 1.6), 7.83 (d, 2H, *J* = 7.8), 7.69 (d, 2H, *J* = 7.8), 7.37 (t, 2H, *J* = 8.2, 7.8), 7.01 (d, 2H, *J* = 7.8), 6.86 (d, 2H, *J* = 8.2), 4.03 (s, 6H), 2.04-1.98 (m, 8H), 1.54 (s, 18H), 1.08 (sext, 8H, *J* = 22.2, 14.7, 7.3), 0.68-0.59 (m, 20H).

^{13}C DEPT135 NMR (100 MHz, $CDCl_3$): δ 165.51 (C), 160.52 (C), 159.10 (CH), 156.24 (C), 155.47 (C), 154.08 (C), 152.43 (C), 142.60 (C), 140.22 (C), 137.46 (C), 130.86 (C), 130.42 (CH), 130.33 (CH), 128.98 (C), 127.93 (C), 127.24 (C), 126.70 (CH), 124.85 (CH), 124.57 (C), 122.85 (C), 122.76 (CH), 117.09 (CH), 114.94 (CH), 113.72 (CH), 113.19 (C), 109.46 (CH), 55.94 (CH), 53.03 (C), 39.99 (CH_2), 35.23 (C), 31.46 (CH), 26.08 (CH_2), 23.04 (CH_2), 13.83 (CH).

Compound ***4***

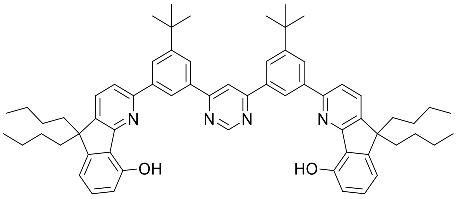

Compound **3** (0.360 g, 0.375 mmol) was added to a round-bottomed flask. Pyridine hydrochloride (0.867 g, 7.50 mmol) was added and the reaction mixture was heated to 250°C under argon for 12 h. The reaction mixture was cooled to room temperature. Water was added and the resulting precipitate was filtered *in vacuo* and washed with water to give the desired product as a grey solid. Yield: 0.243 g, 0.261 mmol, 70%

^{1}H NMR (400 MHz, $CDCl_3$): δ 9.43 (s, 1H), 8.60 (s, 2H), 8.32-8.31 (m, 5H), 7.80-7.74 (m, 4H), 7.34 (t, 2H, J = 7.8), 6.95 (d, 2H, J = 7.3), 6.90 (d, 2H, J = 7.8), 2.07-1.95 (m, 8H), 1.53 (s, 18H), 1.13 (sext, 8H, J = 22.2, 14.7, 7.3), 0.78-0.69 (m, 20H).

^{13}C DEPT135 NMR (100 MHz, $CDCl_3$): δ 165.14 (C), 161.28 (C), 159.26 (CH), 154.81 (C), 154.47 (C), 152.79 (C), 152.46 (C), 142.25 (C), 139.43 (C), 137.47 (C), 131.15 (CH), 131.01 (CH), 126.53 (CH), 124.93 (CH), 124.28 (C), 123.09 (CH), 117.98 (CH), 114.30 (CH), 113.43 (CH), 54.32 (C), 39.46 (CH_2), 35.24 (C), 31.46 (CH_3), 26.25 (CH_2), 23.03 (CH_2), 13.83 (CH_3).

HRMS ($FTMS^+$): for $[M+H]^+$ calculated 931.5885, found 931.5873.

Compound ***5***

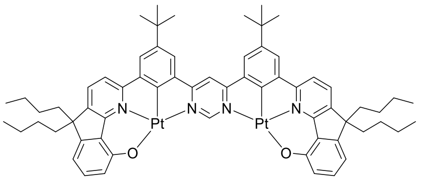

The proligand **4** (0.103 g, 0.110 mmol) was added to a round-bottomed flask. Potassium tetrachloroplatinate (0.114 g, 0.275 mmol) was added. A 9:1 mixture of acetic acid and chloroform (50 mL) was added and the reaction was heated to reflux for 3 days. The mixture was cooled to room temperature and neutralised with a saturated solution of Na_2CO_3. The reaction mixture was then extracted with dichloromethane. The organic phase was separated and dried with $MgSO_4$. The solvent was evaporated to dryness and the crude red residue purified by column chromatography (silica gel, DCM 100:0). The solid obtained was further purified by recrystallisation from methanol to give the desired product as a dark red solid. Yield: 0.0301 g, 0.0228 mmol, 21 %

^{1}H NMR (600 MHz, $CDCl_3$): δ 10.25 (br s, 1H), 8.06 (br s, 1H), 7.95 (d, 2H, J = 7.3), 7.89-7.87 (br m, 4H), 7.62-7.61 (m, 4H), 7.46 (d, 2H, J = 8.2), 6.74 (d, 2H, J = 7.3), 2.13-2.04 (m, 8H), 1.54 (s, 18H), 1.19-1.13 (m, 8H), 0.88-0.80 (m, 4H), 0.76-0.67 (m, 16H).

HRMS ($FTMS^+$): for $[M+H]^+$ calculated 1318.4884, found 1318.4860.

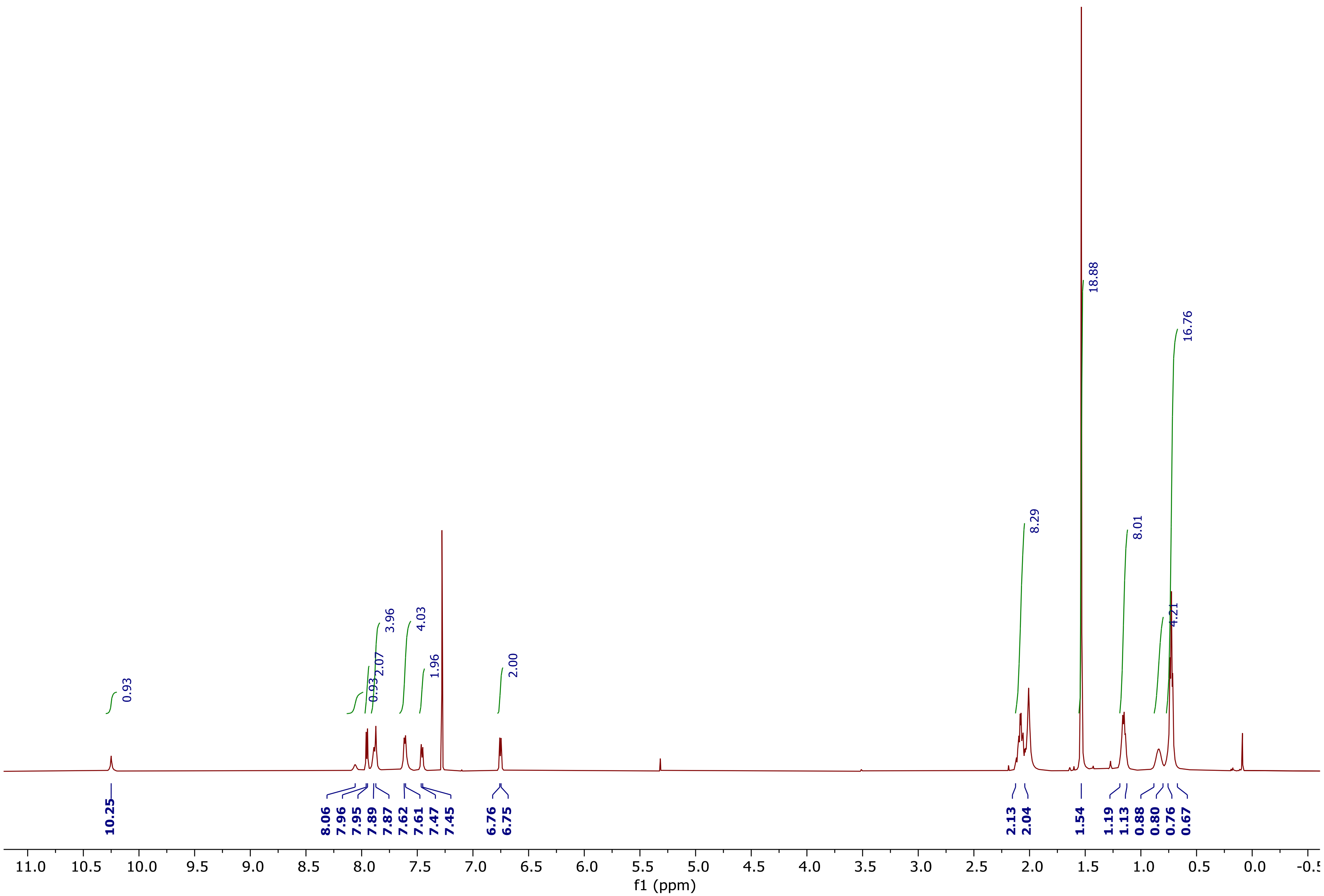

Figure S2.1 The ^{1}H NMR spectrum of di platinum(II) complex **5** in $CDCl_3$.

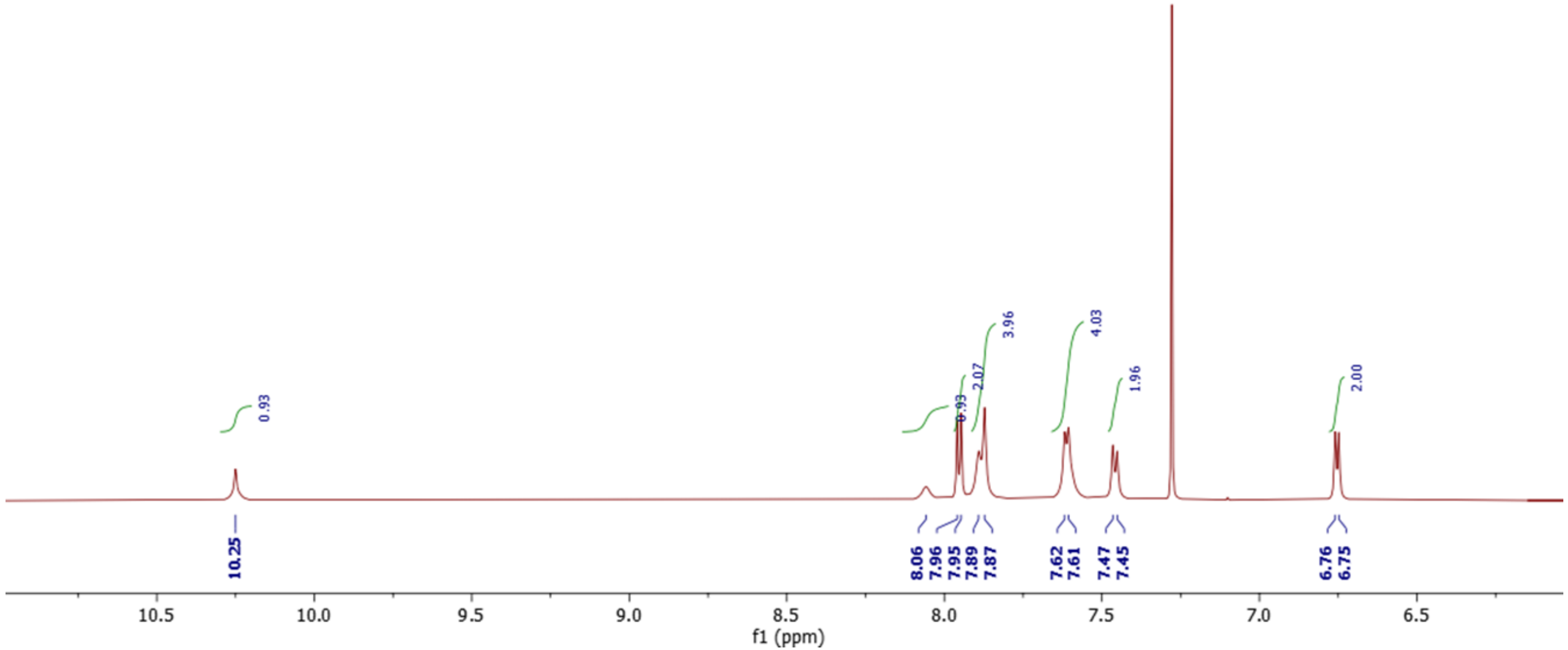

Figure S2.2 The scale up of the aromatic region of the ^{1}H NMR spectrum of di platinum(II) complex **5** in $CDCl_3$. Broadening of the signals of the pyrimidine fragment (at 10.25 and 8.06 ppm) is due to coupling with ^{195}Pt.

3. X-ray diffraction analysis

The X-ray single crystal data for compound **5** have been collected using λMoKα radiation (λ = 0.71073Å) on a Bruker D8Venture diffractometer (Photon100 CMOS detector, IμS-microsource, focusing mirrors) equipped with a Cryostream open-flow nitrogen cryostat (Oxford Cryosystems) at the temperature of 120.0(2)K. The structure was solved by direct method and refined by full-matrix least squares on F^2 for all data using Olex2[17] and SHELXTL[18] software. All non-disordered non-hydrogen atoms were refined in anisotropic approximation; hydrogen atoms were placed in the calculated positions and refined in riding mode. The structure also contains a severely disordered solvent molecule (most probably half of DCM) which could not be reliably modelled and has been taken into account using the MASK procedure of OLEX2 program package. Crystal data and parameters of refinement are listed in Table S3.1 below. Crystallographic data for the structure have been deposited with the Cambridge Crystallographic Data Centre as supplementary publication CCDC-2047145.

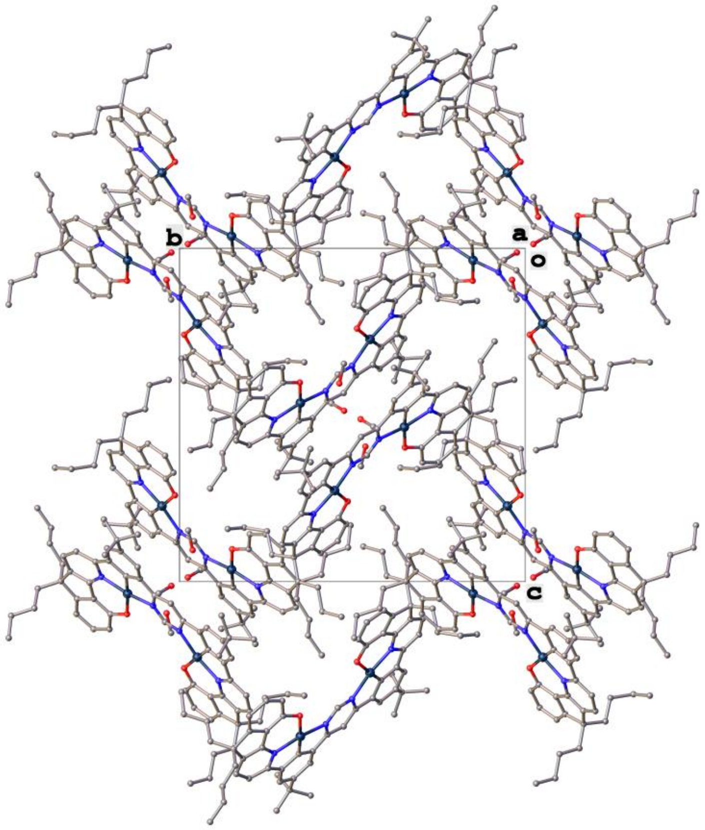

Figure S3.1 Illustrating the packing of molecules of complex **5** in the crystal. Nitrogen atoms in blue, oxygen in red; Pt(II) ions are turqoise spheres.

Table S3.1 Crystal data and structure refinement for **5**.

Identification code	**5**
Empirical formula	$C_{66}H_{78}N_4O_4Pt_2$
Formula weight	1381.50
Temperature/K	120.0
Crystal system	monoclinic
Space group	$P2_1/c$
a/Å	13.3643(14)
b/Å	21.517(2)
c/Å	20.834(2)
α/°	90
β/°	94.905(3)
γ/°	90
Volume/Å^3	5969.0(11)
Z	4
ρ_{calc}g/cm^3	1.537
μ/mm^{-1}	4.732
F(000)	2760.0
Crystal size/mm^3	0.12 × 0.08 × 0.005
Radiation	Mo Kα (λ = 0.71073)
2Θ range for data collection/°	4.222 to 52
Index ranges	$-16 \le h \le 16$, $-26 \le k \le 26$, $-25 \le l \le 25$
Reflections collected	73424
Independent reflections	11726 [R_{int} = 0.2649, R_{sigma} = 0.2217]
Data/restraints/parameters	11726/6/686
Goodness-of-fit on F^2	1.007
Final R indexes [I>=2σ (I)]	R_1 = 0.0762, wR_2 = 0.1231
Final R indexes [all data]	R_1 = 0.1811, wR_2 = 0.1499
Largest diff. peak/hole / e Å^{-3}	0.99 / -1.38

4. Theory

Table S4.1 The energies of the ground and low-lying excited states of complex **5** and **6** calculated at the ground state (S_0) and singlet excited state (S_1) optimised geometries. Note the S_0 energy at S_1 geometry is relative to the S_0 energy at S_0 geometry.

	**5**		**6**	
	**Ground state geometry, eV**	**S_1 excited state geometry, eV**	**Ground state geometry, eV**	**S_1 excited state geometry, eV**
S_0	0.00	0.07	0.00	0.16
S_1	2.20	2.00	2.64	2.38
S_2	2.34	2.16	2.68	2.61
T_1	2.02	1.67	2.31	2.01
T_2	2.17	1.92	2.40	2.39
T_3	2.28	2.24	2.76	2.68

Table S4.2 Transition oscillator strength.

**5**		**6**	
**$S_0 \rightarrow S_1$**	**$S_0 \rightarrow T_1$**	**$S_0 \rightarrow S_1$**	**$S_0 \rightarrow T_1$**
0.30	5×10^{-5}	0.015	6×10^{-6}

5. Photophysics

a) Solution state

It is worth noting that methylcyclohexane is a bad solvent for **5**, thus not very effective in solubilizing **5** molecules and avoiding aggregation completely. Although the emission spectra at $10^{-6} – 10^{-5}$ M remain identical (**Figure S5.1**), a significant change in the absorption spectrum is observed in this concentration range (**Figure S5.2**). The excitation spectrum of **5** emission at 605 nm closely resembles the absorption at low concentrations (**Figure S5.3**). This inevitably indicates that despite the large 83% PLQY in MCH, **5** still forms some kind of aggregate states in this solvent that are non- or hardly luminescent.

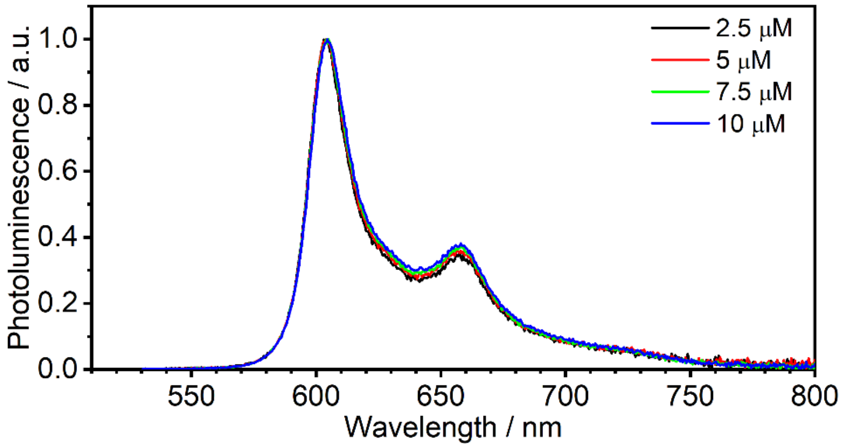

Figure S5.1 Photoluminescence spectra of **5** at various concentrations.

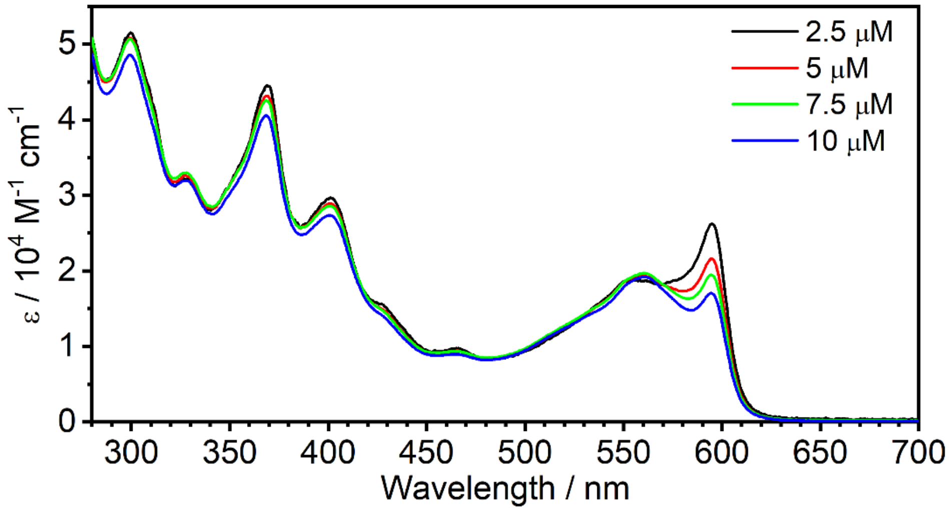

Figure S5.2 Absorption spectra of **5** in methylcyclohexane (MCH) at various concentrations.

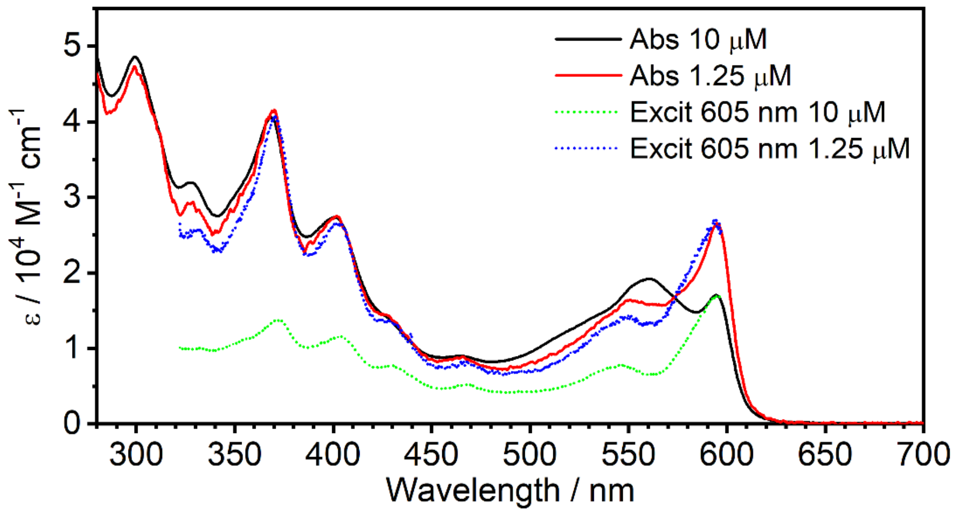

Figure S5.3 Absorption and excitation spectra of **5** in methylcyclohexane (MCH) at two different concentrations. Excitation spectra are normalised to the respective absorption peaks related to the $S_0 \rightarrow S_1$ transition.

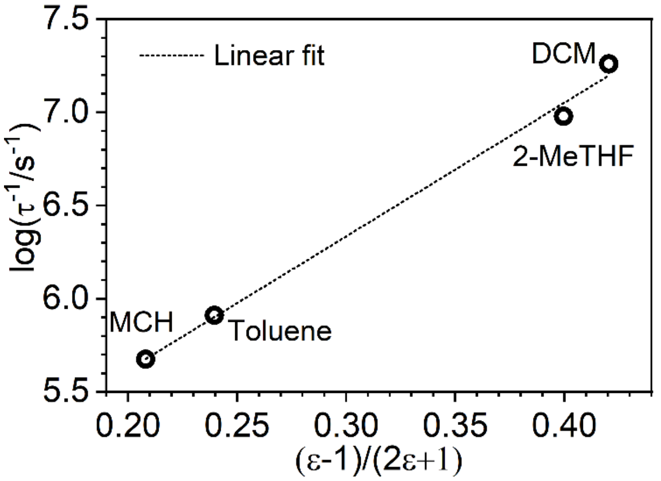

Figure S5.4 Variation of excited state lifetime with solvent polarity; ε - solvent dielectric constant.

b) Solid film (polymer matrix)

Singlet energy in Zeonex (2.15 eV) is smaller than the respective maximum value in TPD:PBD (2.19 eV). In fact, the onset energy of 2.15 eV in Zeonex is recorded from emission spectra at temperature 250 K and below. Fluorescence energy at 295 K in this case is equal to 2.21 eV which occurs due to a high energy shoulder that is present in the spectrum. Such shoulder has been observed previously and assigned to emission occurring from upper vibronic levels of the S_1 state, due to existing thermal equilibrium. Hence, knowing the origin of such shoulder the value of 2.15 eV was used instead.[19]

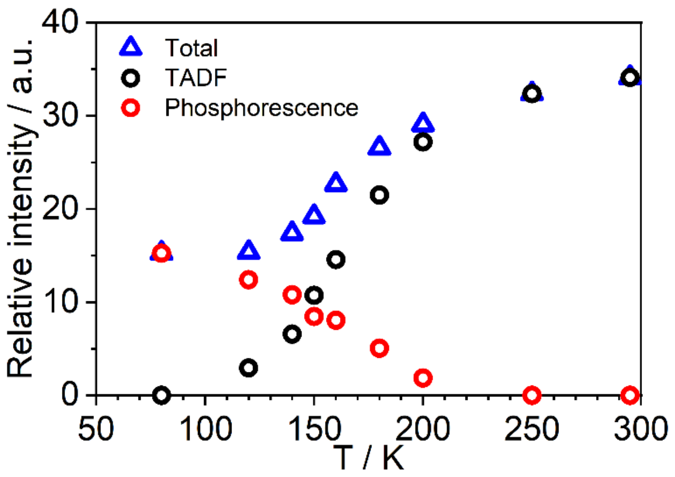

Figure S5.5 Intensity of TADF, phosphorescence, and both combined as a function of temperature in Zeonex film.

Photophysical properties of **5** in Zeonex and polystyrene are identical and no changes to the TADF activation energy or singlet state radiative rate, $k_{PF} = (1.3 \pm 0.4)\times10^7$ s^{-1} can be found (**Figure S5.6**, **Figure S5.7**, and **Figure S5.8**). On the other hand, the spectral S-T gap appears to be slightly larger, $\Delta E_{ST} = 100 \pm 15$ meV. The reason for this discrepancy is the inhomogeneous broadening of the emission spectrum in polystyrene when compared to Zeonex, which is evidenced in the fluorescence of **5** at 295 K. Despite the emission sharing the same vibronic structure (**Figure S5.9** and **Figure S5.10**), the spectrum in polystyrene is significantly broadened and its vibronic structure is not so strongly pronounced as it is in Zeonex or methylcyclohexane. For this reason, we believe the energy of the singlet state determined from the fluorescence onset around room temperature is overestimated. The problem also exists in Zeonex but to a lesser extent. This broadening disappears at lower temperatures (i.e. 200-250

K), and if the onset of the emission at 250 K is used, instead of the 295 K, the $E_a = \Delta E_{ST}$ condition is recovered. It might be that **5** experiences an additional vibrational mode at room temperature that contributes to some molecules emitting from their upper vibronic levels, causing this broadening.[20]

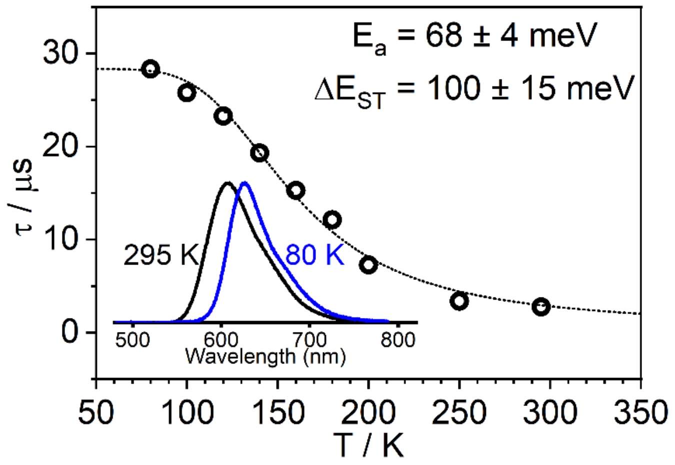

Figure S5.6 Photoluminescence decay lifetime of **5** in polystyrene at various temperatures. The temperature dependence is fitted using equation 1 in the main text.

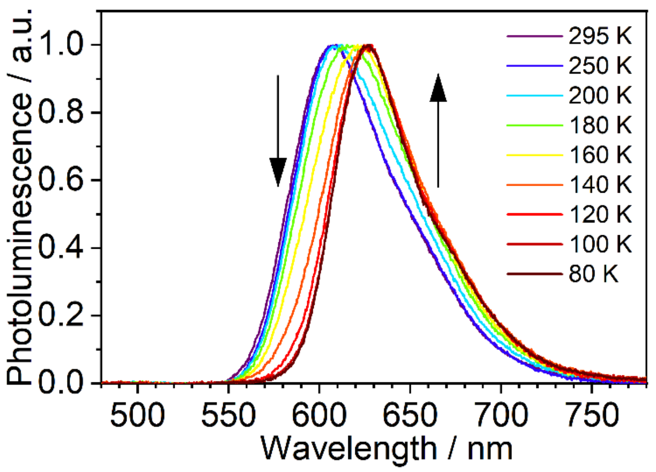

Figure S5.7 Emission spectra of of **5** in polystyrene matrix at various temperatures.

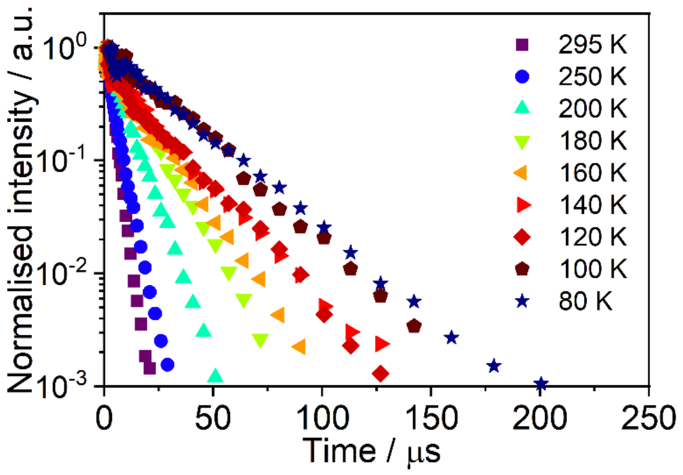

Figure S5.8 Photoluminescence decay of **5** in polystyrene matrix at various temperatures.

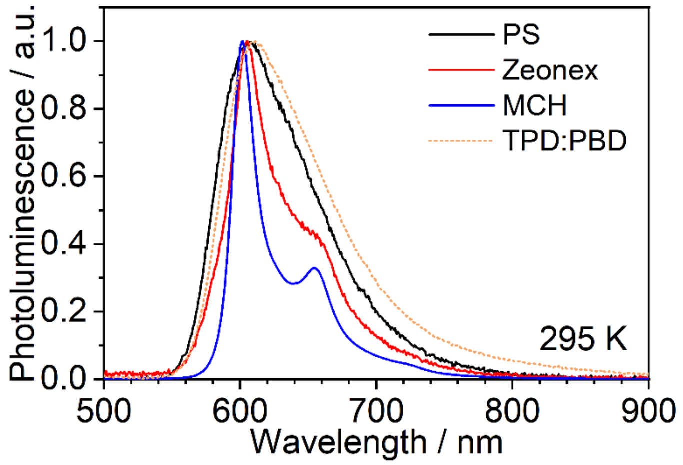

Figure S5.9 Comparison of photoluminescence spectrum of **5** in various media at 295 K.

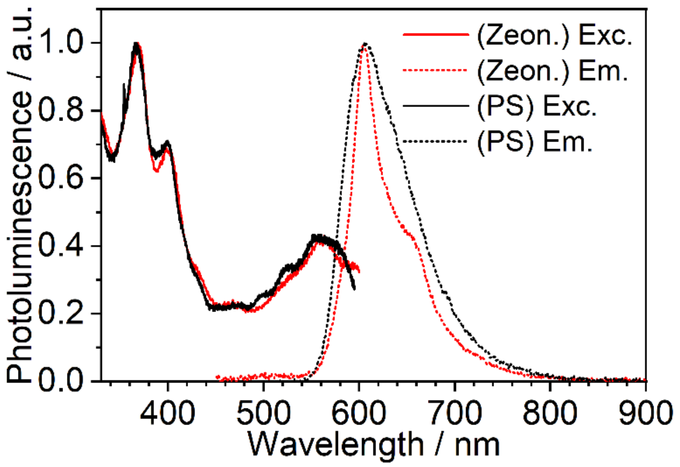

Figure S5.10 Photoluminescence excitation spectrum (continuous line, λ_{em} = 610 nm) and emission spectrum (dotted line) of **5** in polystyrene (black) and Zeonex (red) thin films at 295 K.

c) Studies in an OLED host material

Studies of **5** dispersed in TPD:PBD were carried out using the same composition as that of thin films used in devices. The emission spectrum in this host is shown in **Figure S5.13**; the $\Phi_{PL} = 0.31 \pm 0.05$ is smaller than in a solution, probably due to aggregation. The photoluminescence decay (**Figure S5.11**) is significantly more complex than in solution, Zeonex or polystyrene, which is probably also caused by the higher emitter concentration when compared to those used in the former photophysical studies that were aimed at characterising predominantly isolated molecules (5 % vs 0.1%). The decay shows a weak short-lived fluorescence from the host, followed by longer-lived photoluminescence of **5**. This is observed at every temperature and indicates that the Förster energy transfer from host to guest is not fully complete – a strong indication that a lower dopant concentration is not suitable for OLEDs using this host. The decay of **5** emission is characterized by two main decay components indicating a more heterogenous environment for the emitter: $\tau_1 = 65 \pm 11$ ns (75 %), $\tau_2 = 0.7 \pm 0.1$ μs (25 %) at 295 K and $\tau_1 = 0.46 \pm 0.08$ μs (59 %), $\tau_2 = 8 \pm 1$ μs (41 %) at 80 K. Overall, the shorter τ in the OLED host in relation to the polymer film and solution may indicate that additional quenching processes are active. Note that the host fluorescence lifetime varies from 2.5 ± 0.2 ns at 295 K to 6.1 ± 0.7 ns at 80 K, and thus does not interfere with the luminescence decay of **5**. A third, long-lived component is present at lower temperatures, which may be assigned to traps also occurring in other bicomponent (donor + acceptor) blend hosts[21,22] and in exciplexes.[23,24] The decay does vary with T in a manner qualitatively consistent with TADF

but, due to the complicated nature of the dopant emission, lifetime data were not subject to analysis towards determination of the activation energy.

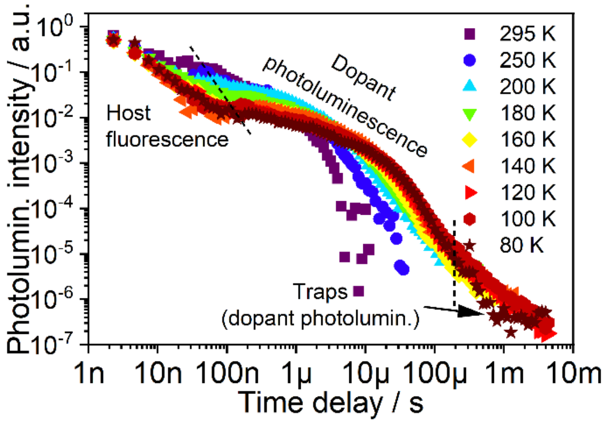

Figure S5.11 Photoluminescence decay of **5** (5% w/w) in TPD:PBD host at various temperatures.

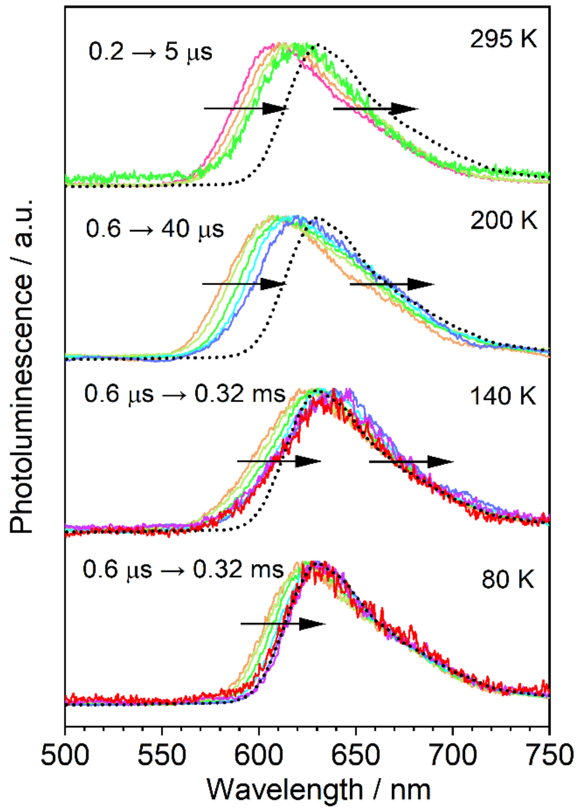

Figure S5.12 Time-resolved spectra of **5** (5% w/w) in TPD:PBD host at various temperatures. Black dotted line represents phosphorescence spectrum at 80 K with 40 μs delay. Note the prompt fluorescence of the host is omitted for clarity.

Figure S5.12 shows the temporal evolution of the emission spectra in TPD:PBD thin film at four different temperatures (the host prompt fluorescence < 0.2 μs is omitted for clarity). Firstly, in clear contrast to experiments recorded at low concentrations (0.1 % w/w) in polymer films, here the emission spectrum red-shifts with time delay at all temperatures. No such spectral drift was observed at low concentrations in the polymer films. This behaviour explains the broadening of the emission spectrum relative to the 0.1% doped Zeonex thin film. Spectral red-shifts can originate from different causes, including the presence of independent emissive states with different lifetimes, in which the states emitting at longer times exhibit emission of lower energy. Such distribution of electronic states is often assigned to a dispersion of the dihedral angle between donor and acceptor units in TADF emitters based on typical donor–acceptor design.[25] However, this effect cannot occur in **5** and such dispersion must originate elsewhere. The most likely cause for the observed spectral shifts is the variation in relative orientation of host-guest dipoles or intermolecular interactions of dopant molecules that occur in the ground state, which may affect the charge-transfer excited state of **5**.[26]

In contrast with the low-concentration studies, the emission spectrum in TPD:PBD {TPD - *N*,*N*'-bis(3-methylphenyl)-*N*,*N*'-bis(phenyl)-benzidine; PBD - 2-(4-biphenyl)-5-(4-*tert*-butylphenyl)-1,3,4-oxadiazole} thin film is not a superposition of just two emissions originating from S_1 and T_1 states, respectively. Instead, the emission attributed to the S_1 state shows a broad distribution of energy, while the emission originating from the T_1 state appears to remain constant in time. This is in agreement with the S_1 and T_1 behaviour in the monometallic analogue[27] **6**, as it appears that both complexes **5** and **6** show an environment-sensitive S_1 (*i.e.*, substantial CT character) and significantly less sensitive T_1 (lower CT character). This phenomenon can be better observed in the time-resolved spectra at 80 K where only residual delayed fluorescence can be seen at early times, 0.6–20 μs, as a blue shoulder of the emission spectrum. This blue shoulder shows a time drift consistent with that observed at higher temperatures. On the other hand, at times > 40 μs, there are no further changes to the spectrum and this emission is assigned to phosphorescence. The singlet states show energy between 2.15-2.19 eV at 295 K, while the triplet energy at 80 K remains constant at 2.07 eV, close to the value obtained in Zeonex (2.09 eV). This gives a range of ΔE_{ST} = 70-120 meV. The simultaneous observation of phosphorescence and delayed fluorescence, at lower temperatures, decaying with different lifetimes, as seen in **Figure S5.12**, is related to molecules with a higher energy singlet (thus experiencing larger ΔE_{ST}) that preferentially decay through phosphorescence, while molecules with lower energy S_1 states receive sufficient thermal energy

to preferentially decay through the singlet state *via* TADF. This is consistent with the reduction of the energy of the delayed fluorescence onset at lower temperatures.

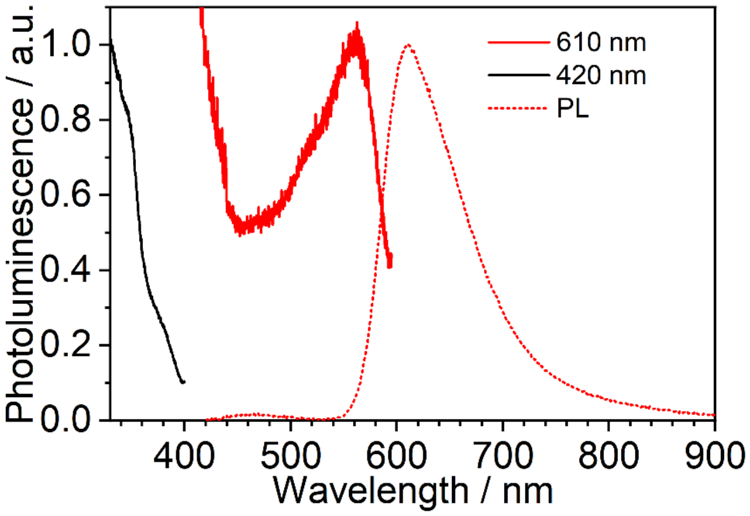

Figure S5.13 Excitation profiles and emission spectrum of **5** in TPD:PBD thin film. Dashed and dotted line represents absorption spectrum in MCH solution showing strong dimer absorption band.

Excitation spectrum of **5** in TPD:PBD thin film (**Figure S5.13**) shows two different profiles: at 610 nm, related to emission of the Pt(II) complex and at 420 nm, related to the emission of the TPD:PBD blend.

6. Electrochemistry

5 shows a typical electrochemical behaviour to many other Pt(II) complexes (**Figure S6.1**). Oxidation of the complex is irreversible due to $d_{Pt(II)}$ orbital admixtures of the central atom to the HOMO. Thus, the metal gives up electron(s) reaching higher oxidation states. I.e. the *onset* oxidation potential of the complex E_{onset}^{ox} = 0.41 V is a result of the ligand not being strongly electron-rich. By using electron-rich ligands HOMO is destabilised leading to a *quasi*-reversible oxidation.[28] On the other hand the ligand stabilises LUMO of the complex, mostly due to its strong electron-withdrawing character that originates from presence of pyrimidine and pyridine moieties. The presence of pyrimidine linker is a crucial factor differentiating **5** from its monometallic analogue[27] as the ligand in the latter has significantly weaker electron-withdrawing properties. This as an effect gives a E_{onset}^{red} = -1.45 V. Consequently, the ionization potential (IP) and electron affinity (EA) of the compound equal to IP = 5.51 eV, EA = 3.65 eV. Thus, electrochemical energy gap of the material E_g^{el} = 1.86 eV.

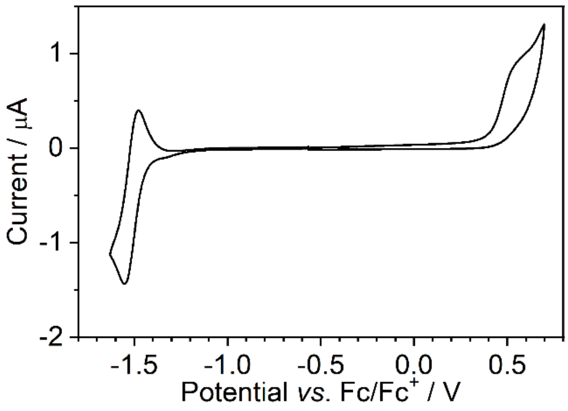

Figure S6.1 Cyclic voltammogram (CV) of **5** (10^{-3} M) in 0.1 M Bu_4NBF_4 in CH_2Cl_2.

7. OLED devices

Exciplex hosts, such as TPD:PBD, have been successfully and commonly used for other metal complexes.[29–31] Moreover, the TPD:PBD mixture is also readily soluble in toluene exhibiting excellent film-forming properties. However, as PBD is an electron-transporting and hole-blocking material, TPD does transport holes, but does not block electrons. This results in very low-efficiency devices if an additional electron-blocking layer is not used.

The percentage doping of the emitter varies usually between 1-20% in typical OLED devices. 5 % doping concentration has found to be a successful approach in solution-processed devices of various kinds.[22,25,28,32] Specifically, in the case of **5**, which was found to form non-emissive aggregates in methylcyclohexane and in solid film (see discussion of the photophysics in film and solution), we aimed the doping level to be maintained as low as possible. However, at a doping level below 5 % there is insufficient amount of dopant to trap all charge carriers, promoting recombination in the host.

8. References

1 F. Neese, *WIREs Comput. Mol. Sci.*, 2012, **2**, 73–78.

2 J. Eng, B. A. Laidlaw and T. J. Penfold, *J. Comput. Chem.*, 2019, jcc.25868.

3 H. Iikura, T. Tsuneda, T. Yanai and K. Hirao, *J. Chem. Phys.*, 2001, **115**, 3540–3544.

4 C. Fonseca Guerra, J. G. Snijders, G. te Velde and E. J. Baerends, *Theor. Chem. Accounts Theory, Comput. Model. (Theoretica Chim. Acta)*, 1998, **99**, 391–403.

5 S. J. A. van Gisbergen, J. G. Snijders and E. J. Baerends, *Comput. Phys. Commun.*, 1999, **118**, 119–138.

6 ADF2009.01, SCM, https://www.scm.com/.

7 F. Wang and T. Ziegler, *J. Chem. Phys.*, 2005, **123**, 154102.

8 E. van Lenthe, E. J. Baerends and J. G. Snijders, *J. Chem. Phys.*, 1993, **99**, 4597–4610.

9 E. van Lenthe, E. J. Baerends and J. G. Snijders, *J. Chem. Phys.*, 1994, **101**, 9783–9792.

10 P. Data, P. Pander, M. Lapkowski, A. Swist, J. Soloducho, R. R. Reghu and J. V. Grazulevicius, *Electrochim. Acta*, 2014, **128**, 430–438.

11 P. Pander, P. Data, R. Turczyn, M. Lapkowski, A. Swist, J. Soloducho and A. P. Monkman, *Electrochim. Acta*, 2016, **210**, 773–782.

12 C. M. Cardona, W. Li, A. E. Kaifer, D. Stockdale and G. C. Bazan, *Adv. Mater.*, 2011, **23**, 2367–2371.

13 J.-L. Bredas, *Mater. Horiz.*, 2014, **1**, 17–19.

14 P. Pander, P. Data and F. B. Dias, *J. Vis. Exp.*, , DOI:10.3791/56614.

15 D. de Sa Pereira, A. P. Monkman and P. Data, *J. Vis. Exp.*, , DOI:10.3791/56593.

16 The University of Hong Kong, WO/2013/152727, 2013.

17 O. V. Dolomanov, L. J. Bourhis, R. J. Gildea, J. A. K. Howard and H. Puschmann, *J. Appl. Crystallogr.*, 2009, **42**, 339–341.

18 G. M. Sheldrick, *Acta Crystallogr. Sect. A Found. Crystallogr.*, 2008, **64**, 112–122.

19 P. Pander, R. Motyka, P. Zassowski, M. K. Etherington, D. Varsano, T. J. da Silva, M. J. Caldas, P. Data and A. P. Monkman, *J. Phys. Chem. C*, 2018, **122**, 23934–23942.

20 H. F. Higginbotham, P. Pander, R. Rybakiewicz, M. K. Etherington, S. Maniam, M. Zagorska, A. Pron, A. P. Monkman and P. Data, *J. Mater. Chem. C*, 2018, **6**, 8219–8225.

21 V. Jankus, K. Abdullah, G. C. Griffiths, H. Al-Attar, Y. Zheng, M. R. Bryce and A. P. Monkman, *Org. Electron.*, 2015, **20**, 97–102.

22 P. Pander, R. Bulmer, R. Martinscroft, S. Thompson, F. W. Lewis, T. J. Penfold, F. B. Dias and V. N. Kozhevnikov, *Inorg. Chem.*, 2018, **57**, 3825–3832.

23 P. Pander, S. Gogoc, M. Colella, P. Data and F. B. Dias, *ACS Appl. Mater. Interfaces*, 2018, **10**, 28796–28802.

24 M. Chapran, P. Pander, M. Vasylieva, G. Wiosna-Salyga, J. Ulanski, F. B. Dias and P. Data, *ACS Appl. Mater. Interfaces*, 2019, **11**, 13460–13471.

25 R. Pashazadeh, P. Pander, A. Lazauskas, F. B. Dias and J. V. Grazulevicius, *J. Phys. Chem. Lett.*, 2018, **9**, 1172–1177.

26 M. Tanaka, H. Noda, H. Nakanotani and C. Adachi, *Appl. Phys. Lett.*, 2020, **116**, 023302.

27 P. K. Chow, C. Ma, W. P. To, G. S. M. Tong, S. L. Lai, S. C. F. Kui, W. M. Kwok and C. M. Che, *Angew. Chemie - Int. Ed.*, 2013, **52**, 11775–11779.

28 M. Z. Shafikov, R. Daniels, P. Pander, F. B. Dias, J. A. G. Williams and V. N. Kozhevnikov, *ACS Appl. Mater. Interfaces*, 2019, **11**, 8182–8193.

29 J. H. Lee, S. H. Cheng, S. J. Yoo, H. Shin, J. H. Chang, C. I. Wu, K. T. Wong and J. J. Kim, *Adv. Funct. Mater.*, 2015, **25**, 361–366.

30 Y. Park, S. Lee, K. Kim, S. Kim, J. Lee and J. Kim, 2013, 4914–4920.

31 S. Lee, K. Kim, S. Yoo, Y. Park and J. Kim, 2013, **8829**, 1–7.

32 A. Klimash, P. Pander, W. T. Klooster, S. J. Coles, P. Data, F. B. Dias and P. J. Skabara, *J. Mater. Chem. C*, 2018, **6**, 10557–10568.
